# tCRISPRi: tunable and reversible, one-step control of gene expression

**DOI:** 10.1038/srep39076

**Published:** 2016-12-20

**Authors:** Xin-tian Li, Yonggun Jun, Michael J. Erickstad, Steven D. Brown, Adam Parks, Donald L. Court, Suckjoon Jun

**Affiliations:** 1 Section of Molecular Biology, Division of Biological Sciences and Department of Physics, UC San Diego, La Jolla, CA 92093, USA; 2 RNA Biology Laboratory, Center for Cancer Research, National Cancer Institute at Frederick, Frederick, Maryland 21702, USA

## Abstract

The ability to control the level of gene expression is a major quest in biology. A widely used approach employs deletion of a nonessential gene of interest (knockout), or multi-step recombineering to move a gene of interest under a repressible promoter (knockdown). However, these genetic methods are laborious, and limited for quantitative study. Here, we report a tunable CRISPR-cas system, “tCRISPRi”, for precise and continuous titration of gene expression by more than 30-fold. Our tCRISPRi system employs various previous advancements into a single strain: (1) We constructed a new strain containing a tunable arabinose operon promoter *P*_BAD_ to quantitatively control the expression of CRISPR-(d)Cas protein over two orders of magnitude in a plasmid-free system. (2) tCRISPRi is reversible, and gene expression is repressed under knockdown conditions. (3) tCRISPRi shows significantly less than 10% leaky expression. (4) Most important from a practical perspective, construction of tCRISPRi to target a new gene requires only one-step of oligo recombineering. Our results show that tCRISPRi, in combination with recombineering, provides a simple and easy-to-implement tool for gene expression control, and is ideally suited for construction of both individual strains and high-throughput tunable knockdown libraries.

CRISPR-Cas is an RNA-directed immune system in prokaryotes, which consists of CRISPR (Clustered Regularly Interspaced Short Palindromic Repeats) and CRISPR-associated proteins, Cas. There are three major types of CRISPR-Cas systems based on their evolutionary relationships^1^,^2^,^3^,^4^,^5^. In the Streptococcus pyogenes Type II CRISPR-Cas system, the large single protein Cas9 has RuvC-like and HNH endonuclease domains that are responsible for cleavage of the target DNA to cause double-strand breaks^3^,^4^,^6^,^7^,^8^,^9^. A nuclease-deficient Cas9 (dCas9) having mutations in both HNH and RuvC-like nuclease domains cannot cut DNA^6^,^10^. Instead, dCas9 binds tightly to the target DNA and thereby interferes with transcription^11^,^12^. dCas9 can transiently or stably control gene expression level without changing the gene sequence itself^11^,^13^,^14^. Specific guide RNAs (gRNA), direct the activity of Cas9 to homologous targets for recognition and binding^2^,^3^. A recently developed synthetic system eliminates *in vivo* RNA processing steps that are typically required to generate guide RNAs^3^,^11^. These features of Cas9 make it a potent tool for CRISPR-mediated interference (CRISPRi) as described in recent reviews^8^,^9^,^11^.

While the precision and versatility of CRISPR Cas9 is powerful for genomic DNA editing and gene regulation, many CRISPR Cas9 systems are hampered by toxicity caused by constitutive expression of Cas9^15^,^16^,^17^,^18^. In principle, this problem could be solved by expressing Cas9 under an idealized inducible and tunable promoter. This would allow precise regulation of the expression level of Cas9 in a dose-dependent manner, uniformly throughout the whole cell population. Unfortunately, many commonly used bacterial inducible promoters operate as “on/off” switches, and lack a rheostat-like function that would allow such tunable gene expression^19^,^20^. Recently, Gross and colleagues have expressed the dCas9 under a xylose-inducible promoter to knockdown essential genes in *Bacillus subtilis*, and characterized the network structure of essential genes based on their functional analysis^21^.

A second practical problem for using CRISPR-(d)Cas9 systems for high-throughput experiments is that strain constructions to target new genes are time consuming. Current methods for sgRNA construction mainly rely on plasmid-based molecular cloning techniques, followed by transformation. However, plasmids are typically present in multiple copies, which make them less than ideal when precise quantification is required^22^,^23^. Plasmids also contain their own genes and require selection by drugs for maintenance. Thus, multi-copy plasmid-based systems can inhibit general cell growth and gene expression, with significant effects on cellular physiology^22^.

Here, we develop a tunable CRISPRi (“tCRISPRi”) that can achieve over 30-fold repression for most essential and nonessential genes (see Fig. 1 for a schematic diagram). We also engineered the bacterial genome so that the *P*_BAD_ promoter could be induced with arabinose in a dose-dependent system without bimodal expressions^19^,^20^ showing minimal leakiness in the absence of arabinose. The expression of dCas9 responded in a linear fashion with respect to addition of external arabinose. Importantly, this strain requires only one single-strand oligo recombineering step to insert the desired sgRNA sequence into the chromosome to allow its constitutive expression.

Here we describe the construction of the tCRISPRi strain and use it to target dCas9 to a set of essential and nonessential genes. Their repression by dCas9 was characterized quantitatively. The most important goal of this work is to implement various developments in bacterial genetics into a single standard *E. coli* strain to enable plasmid-free, single-step construction of a tCRISPRi system for precise control of gene expression of both essential and nonessential genes.

## Results

### Establishment of *P*_BAD_-*dcas9* as a plasmid-free arabinose tunable system

A standard method to control gene expression is to clone the gene under an inducible promoter. Unfortunately, most bacterial inducible promoters behave as an “on/off” switch, resulting in bimodal distributions with a population of cells either uninduced or fully induced^19^,^20^,^24^,^25^,^26^. To solve this problem, we have developed an *E. coli* strain containing a tunable arabinose operon promoter *P*_BAD_, which has a wide dynamic range with graded control by arabinose inducer (hereafter, we use arabinose for inducer).

To make the dose-dependent inducible *P*_BAD_ promoter, we eliminated arabinose transporter genes, including *araE* and *araFGH*^19^. The *araBAD* operon was also replaced either by *msfGFP* or *dcas9* in our experiments, which prevents any catabolism of the arabinose used for induction. We next introduced a point mutation in the lactose transporter gene, *lacY* A177C, so that arabinose can freely diffuse into the cell through the mutated transporter^19^. We also deleted *lacI*, the *lac* repressor gene to ensure constitutive expression of LacY A177C. This entire system, including the promoter *P*_BAD_ controlling the expression of *dcas9*, is directly integrated into the genome without relying on any plasmids. See Fig. 2a for the design of the strain. See Supplementary information in Figures S8–S9 for detailed information about the strain construction including the replacement of the *araBAD* genes with *dcas9*.

### Tunable expression of *P*_BAD_ shows over two orders of magnitude dynamic range

We used quantitative fluorescence microscopy to characterize the behavior of *P*_BAD_ in our modified strain. We confirmed that *P*_BAD_ shows linear expression with respect to the arabinose concentration without a bimodal distribution. For this, we replaced *araBAD* with *msfGFP* and measured the expression level of msfGFP. Elimination of *araBAD* prevents metabolism of the arabinose. We expressed *msfGFP* under the *P*_BAD_ promoter at different arabinose concentrations. At each arabinose concentration, the distribution of fluorescence was well described by gamma distribution^27^, with a coefficient of variance (CV) around 0.2 in the linear regime (Fig. 2b). This is in stark contrast to bimodal distributions expected for a typical “on/off” switch-like promoter such as the wild type strain with *P*_BAD_^20^ or *P*_LAC_^26^.

The *P*_BAD_ promoter in our modified strain shows a dynamic range over two orders of magnitude. In particular, the response of *P*_BAD_ is linear for more than one order of magnitude at arabinose concentrations between 0.01% and 0.1%. Thus, our *P*_BAD_ promoter is tunable and well suited for experiments requiring quantitative titration of gene expression.

We next asked if we can turn off *P*_BAD_ by simply removing the inducer from the culture. *P*_BAD_ expression is indeed reversible as expected (Fig. 2d). As we washed the cells (twice), the fluorescence level of the cells immediately started to decrease exponentially (Fig. 2d, inset). The immediate decrease of the fluorescence indicates that *P*_BAD_ responds within minutes to changes in arabinose concentration. The rate of fluorescence signal decrease is limited by protein dilution, which is caused by cell growth and not by fluorescence protein degradation^11^.

### Construction of tCRISPRi, a tunable repression system

The tCRISPRi strains express dCas9 from *P*_BAD_ and sgRNA from a constitutive promoter. Each of these systems is in single copy on the chromosome. Construction of a new tCRISPRi strain requires only one recombineering step, as described in Fig. 3 and Supplementary Information. The Cas9-binding RNA structure and transcription terminator of the sgRNA are already encoded within the chromosome, and are linked to a counter-selectable marker, *tet*-*sacB*^28^. Once the homology targeting sgRNA sequence is inserted by replacing *tet*-*sacB*, down-regulation of the targeted gene is accomplished by adding arabinose to the culture medium. See Materials and Methods for more information.

### tCRISPRi shows more than a 30-fold knockdown dynamic range

We validated tCRISPRi using two independent methods. First, we repressed the expression of the yellow fluorescent protein (YFP) (Fig. 4b, Figure S1). The dynamic range of repression of YFP was about 10-fold for [arabinose] = 0.001–0.1%. In addition, the distribution of YFP per cell was well-described by a gamma distribution (Fig. 4b, inset), consistent with the linear response of *P*_BAD_ described above (Fig. 2b).

Next, we tested repression of LacZ by tCRISPRi over a range of [arabinose] = 0.002–2.0%, and observed increasing repression of LacZ up to 32-fold as measured by β-galactosidase (Fig. 4c). Repression of *lacZ* expression also means that *lacY*, just beyond *lacZ* in the *lac* operon will also be repressed. The shoulder of ß-gal units at [ara] = 0.05% in Fig. 4c is likely due to reduced arabinose uptake due to a polarity effect of *lacZ* upon *lacY* A177C.

### Repression by tCRISPRi is precise, tunable and reversible

We also verified that repression by tCRISPRi is precise, tunable and reversible (Figs 4b and 3d). We measured the level of dCas9 by comparing the targeted level of YFP using a tCRISPRi strain SJ_XTL174 without induction of dCas9 and that of a control strain SJ_XTL427, which lacks dCas9 (see Supplementary Information) (Table S1, Fig. 4b). We found that the leaky expression is approximately 7.5%, which is significantly lower than found in a recent study for *B. subtilis* (Fig. 4b)^21^,^29^.

Repression by tCRISPRi is reversible (Fig. 4d). To show this, we expressed dCas9 using [arabinose] = 0.1%. The level of YFP dropped to a steady-state level after ~3 hours of growth (Fig. 4d). After 2× washing and regrowth in fresh medium without arabinose (the vertical dashed line in Fig. 4d), the YFP level started to increase again and reached 50% of the initial fluorescence level approximately after 3 hours. After total ~10 hours of exponential growth, cells fully regained their initial fluorescence.

### tCRISPRi strains are physiologically robust

The genetic modifications introduced in the tCRISPRi strains may have limited effects on their applicability. We performed multiple growth experiments in different growth media and tested whether the tCRISPRi strains satisfy the growth law. That is, physiologically robust *E. coli* strains should exhibit an exponential dependence of the average cell size on the nutrient-imposed growth rate^30^. The tCRISPRi strains indeed showed the expected exponential behavior even at [arabinose] = 0.1%, where *yfp* is significantly repressed (Fig. 4e). Furthermore, during the 21-hour time-course experiment (Fig. 4d), cells maintained constant growth rate with a doubling time of 32 min during CRISPR interference (13 generations) and recovery after withdrawing arabinose induction (16 generations). We thus found that the physiology of the tCRISPRi strains can be robust.

A generic limitation of using *P*_BAD_ or *P*_LAC_ systems is catabolite repression caused by glucose and certain other sugars in the growth medium^31^,^32^. Indeed, when glucose was added to the medium, the expression level of *yfp* did not change significantly at [arabinose] = 0.1%. Certain carbon sources we tested (glycerol, sorbitol, and mannose), with or without other nutrient supplements, did not impose such a severe limitation on arabinose induction (Fig. 4e,f) and therefore were suitable to study tunable gene expression. Furthermore, our tCRISRPi strain can be used with other inducible promoters. For example, we expressed red fluorescent proteins using a pTet promoter in our tCRISRPi strain, and the pTet promoter showed an expected on/off behavior^33^ (Figure S7).

### Knockdown of essential and nonessential genes using tCRISPRi

We tested the applicability of tCRISPRi for several essential and nonessential genes whose function ranges from DNA replication to cell division. They are three essential genes *rpoB, dnaG*, and *ftsZ*, and three nonessential genes *mCherry, lexA* and *lacZ*. To quantify the level of knockdown by tCRISPRi, we employed a msfGFP fluorescent transcription reporter system.

For the six genes tested here, we observed as much as 32-fold inhibition, with several genes in the range of 2- to 3-fold inhibition. More specifically, for all three essential genes *rpoB, dnaG, ftsZ*, we observed up to 14-fold knockdown in gene expression. The knockdown of these genes caused significant increase in cell size (Fig. 5c). For the non-essential genes *mCherry, lexA* and *lacZ*, the expression level decreased up to 32-fold, and neither the growth rates nor the cell size were affected by the knockdown (Fig. 5c). Note that in the SJ_XTL228 (*lexA* tCRISPRi) culture induced for dCas9, *lexA* transcripts are suppressed by approximately 2 folds. This relatively weak suppression is likely due to LexA’s autoregulation of its own expression. It acts as its own repressor and therefore when protein LexA expression is shut down the *lexA* operon would become active because the LexA repression of itself would be reduced. Thus, genes which show poor inhibition by tCRISPRi may reveal those genes, which are autoregulated.

These results show that the tCRISPRi system can be used for graded suppression of gene expression for both essential and nonessential genes from their wild-type level with minimal leaky expression (Fig. 5c).

### Case study: knockdown of FtsZ

The tubulin homologue FtsZ is essential for cell division in bacteria^34^, and its suppression shows clear phenotypic changes such as filamentation. FtsZ assembles into a septal Z ring at mid-cell, the site of cell division in *E. coli*^34^,^35^.

We constructed a *ftsZ* tCRISPRi strain as described above, and gradually reduced the expression level of FtsZ from its wild-type level (Figs 5c and 6a). At low induction, the average cell size increased, in agreement with previous studies^36^,^37^ (Fig. 6b). However, unexpectedly, this increase in average size was not due to uniform size increase of all cells, but due to the formation of a *subpopulation* of cells that filamented (Fig. 6c). As the level of repression increased, the division of virtually all cells was gradually halted, leading to broad distributions in cell size. At the highest induction, all cells filamented and stopped growing. As arabinose was removed from the cell culture, and washed away from the cells, all cells resumed division and cell sizes returned to normal (Fig. 6d), again confirming the reversibility of tCRISPRi.

**Figure 1:**
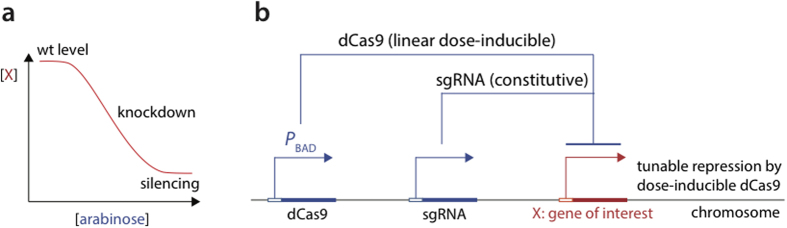
Schematic illustration of tunable repression by tCRISPRi. (**a**) desired gene expression control by tunable tCRISPRi. (**b**) Design strategy of tCRISPRi. dCas9 is expressed by the *P*_BAD_ promoter, whereas sgRNA is constitutively expressed. The level of expression of gene X is controlled by the expression level of dCas9.

Our results are in agreement with previous studies, which used an inducible promoter to modulate the expression level of *ftsZ*^38^,^39^,^40^,^41^. The new insight in the present study is that only subpopulations of cells show delayed cell division at weak suppression of FtsZ. This is unlikely due to any bimodal distribution of arabinose induction by the *P*_BAD_ promoter, since the removal of arabinose transporters in our strain eliminates the feed-forward switch for induction (Fig. 1b). While a detailed investigation of the molecular mechanism of FtsZ expression and its downstream effects is beyond the scope of the present study, our observation warrants further investigation and shows that tCRISPRi and single-cell methods are a powerful combination for obtaining new insights to problems.

### Off-target Analysis by RNA-Seq and mismatch sgRNAs

Gene knockdown must be specific to have high utility. We assessed the specificity of the tCRISPRi system using two different methods: RNA-Seq for transcriptome and mismatch sgRNAs.

#### RNA-Seq for four tCRISPRi strains with sgRNA against yfp, lexA, rpoB, and sacB

We studied the uninduced and induced conditions of the following four strains: SJ_XTL174 (*yfp* tCRISPRi), SJ_XTL228 (*lexA* tCRISPRi), SJ_XTL320 (*rpoB* tCRISPRi), SJ_XTL454 (*sacB* tCRISPRi). The transcription factor gene *lexA* was selected to test the response of cells to a well-established proteomic change; *rpoB* was selected as an essential gene representative, which has been studied with only limited tools. SJ_XTL454 (*sacB* tCRISPRi) is a control strain in which the *sacB-*specific sgRNA does not target any genomic region (*i.e*., the *sacB* gene is not present in the genome). Cells were induced at [arabinose] = 0.2% w/v for all cultures except SJ_XTL320 (*rpoB* tCRISPRi) strain, which was limited to [arabinose] = 0.025% due to growth arrest at higher concentrations.

In all cases, the transcripts for the targeted genes are among the most-reduced of all transcripts in the cultures induced for dCas9 (Fig. 7a). To identify which, if any, of these more-altered transcripts was the result of off-target suppression, we identified genes with potential off-target binding sites for comparison. The first subset consists of genes which have a PAM dinucleotide (CC plus CT/TC) coupled with an exact match to the first 7 nucleotides (region I) of the sgRNA. In all four strains, the subset of potential off-target genes had distributions which were not significantly different from the global distribution of gene expression changes. For all three strains in which we expected targeted gene suppression, the on-target gene was the most repressed gene of the exact region I match. In a second subset composed of genes with the same PAM dinucleotides and up to 2 mismatches in the 12 nucleotides of regions I + II, both *yfp* and *rpoB* were the most repressed genes in their subsets (Figure S4a, red horizontal bars, *p* > 0.5; Table S4, green highlight). In the SJ_XTL228 (*lexA* tCRISPRi) strain, *lexA* was the second most-repressed gene in its region I + II subset, exceeded only by *allS*. This is not unexpected as targeting *lexA* causes LexA expression to be induced, minimizing the targeted inhibition as discussed previously. In the examination of the potential off-target matches with a perfect match to a region I of the sgRNA, we find no relationship between the Hamming distance of the off-target candidate and the repression level in the induced culture (Figure S3a).

Among the most abundant off-target matches identified by GUIDE-Seq are those which contain 3 or 4 mismatches in the entire 20 nucleotides of the sgRNA^42^. No such matches exist for targets with 3 nt differences in the *E. coli* genome for the 4 sgRNAs examined by RNA-Seq. For the subsets of off-target matches with 4 nt differences, only the *lexA* sgRNA had any matches: a single gene (*murD*) which is underrepresented by only 20% in the induced SJ_XTL228 (*lexA* tCRISPRi) sample, a change which is not reflected by other genes in the same operon (Figure S2, green points).

Based on our analysis, we believe that any non-specific changes in gene expression (e.g. *allS* or *murD*) observed here are a result of biological phenomena or technical noise which are not related to the dCas9-sgRNA complex interacting with genomic DNA. In each of the four induced strains, *allS* is at least 2-fold repressed and the *allABCDE* genes are inconsistently altered in each of the four *dCas9*-induced samples versus the uninduced controls (Figure S2, red circles). The sequence of the match in *allS* has only 11/20 matching nucleotides whereas the next most-repressed gene after *lexA, hypB*, has 14/20 matching nucleotides including 10/11 which are also matching in the *allS* sequence. This level of off-target tolerance, is in fact a direct of effect of dCas9 binding, would dramatically exceed that which has been observed in organisms with more abundant and more similar off-target match sites.

#### Analysis of knockdown level with mismatch sgRNAs

To further test specificity of the tCRISPRi system, we studied the level of repression of YFP in the presence of mismatch sequences between the sgRNA and the target gene. To this end, we introduced an increasing number of wobble mutations, from 0 to 5, directly in the *yfp* gene. No mutation was introduced to the sgRNA itself so that the secondary structure of sgRNA remains unchanged.

We observed a decrease in the effect of tCRISPRi as the number of mismatches increased. For normal *yfp* sgRNA with no mismatch, the level of knockdown was approximately 10-fold (Fig. 4b). With 2 to 3 mismatches, the knockdown level was reduced to ~2-fold. By 5 mismatches, the sgRNA completely lost its inhibitory effect against *yfp* (Figure S6).

### Comparison with previous works

Morgan-Kiss *et al*. developed the plasmid-based, dose-inducible promoter *P*_BAD_^19^. Their system allows tunable expression of a protein from the *P*_BAD_ promoter, dependent upon arabinose levels. Their strain expressed a mutant lactose transporter, LacY A177C, from a plasmid. The arabinose transporter genes *araE*201 *araFGH*::*kan* were inactive in the strain. Their strain has two copies of *lacY*; the wild-type *lacY* on the chromosome and *lacY A177C* on a plasmid. The LacY A177C function allows arabinose to freely diffuse into the cell, and thus, the *P*_BAD_ induction level is precisely controlled by the concentration of the supplied arabinose in the medium^19^.

Our tCRISPRi strain contains only the mutant gene *lacY* A177C^19^, which is expressed from the *lac* operon constitutively because the *lacI* repressor gene is deleted. Our strain also has gene deletions of *araE*, and *araFGH*. The *lacY* mutation in this strain expresses LacY A177C, which is the only arabinose transporter in the cell allowing for better control of the *P*_BAD_ promoter and tunable repression by tCRISPRi.

A recent study by Peters *et al*. showed the power of CRISPR-based knockdown methods for studying essential genes in *B. subtilis*^21^. Their sgRNA libraries were cloned via inverse PCR, and dCas9 was under an xylose-inducible promoter. In contrast, our tCRISPRi system for *E. coli* uses one-step recombineering to make a tCRISPRi strain. The *P*_BAD_ promoter in the present work shows about 7.5% leaky expression, whereas the *B. subtilis* CRISPRi shows approximately 33% leakiness. Another important pioneering CRISPRi system was designed by the Marraffini group, who used a plasmid-based system^12^. We compare our tCRISPRi with these other two systems in Table 1.

## Discussion

The tunable tCRISPRi system alleviates most of the known problems of plasmid-based expression methods, and can be immediately used to construct libraries of sgRNAs that can complement the Keio collection^43^ by targeting both essential and nonessential genes. Construction of an an sgRNA or a complete library of sgRNAs requires an oligonucleotide recombination step at a specifically designed locus (Fig. 3). Since the recombineering described here yields many recombinants per reaction (10^5^ recombinants per 10^8^ viable cells), it can efficiently generate a CRISPRi library, or optimize gene targeting and screen for genes or mutants that yield a particular phenotype when knocked down. Upon tunable induction, we can study effects of a wide range of expression levels of any gene of interest, from moderate to severe knockdown of a targeted gene’s transcription. To enable researchers to quickly adapt this strain for their own experiments, we have designed sgRNAs and the oligonucleotides required to generate them by recombineering for nearly the entire *E. coli* genome (sequences, Table S4; design methodology, Supplementary Experimental Methods).

In addition to gene repression by tCRISPRi, and it is known that dCas9 can mediate gene activation as a fusion protein of dCas9 and the ω subunit of RNA polymerase^12^. Recently, scaffold RNAs designed to repress and activate genes simultaneously in mammalian cells has been reported^44^. By introducing the dCas9-ω fusion gene under control of *P*_BAD_ we can quantitatively control dCas9-ω, and therefore induction of the target gene promoter. This technique may effectively increase gene expression, in contrast to the inhibition of expression described above. We again foresee the extension of this technology to include manipulation of multiple genes by designing multiple sgRNAs, which may be used in studying and optimizing metabolic pathways.

The tCRISPRi technique that we have described represents a significant savings in time, effort, and expense. Elimination of cloning steps, and streamlining sgRNA construction down to a single single-strand-oligonucleotide have enabled rapid and simple development of new CRISPR targets. Others have adapted recombineering technologies for use in high throughput applications, and similarly, this system may easily be adapted for high throughput CRISPR reagent development and analysis^45^. The introduction of a tunable *P*_BAD_ strain allows exquisite control of targeted genes and, incorporating various (d)Cas9 modifications^46^, this versatile technique is an ideal system for addressing a wide variety of scientific inquiries and industrial applications.

## Materials and Methods

### Strains and plasmids

All bacterial strains used in this work are listed in Table S1. Plasmid pdCas9 (Addgene# 46569)^12^, plasmid PgRNA-bacterial (Addgene # 44251)^11^ and plasmid pC008 (Addgene# 79157)^33^ are from Addgene, plasmid pDHL1029 and pDHL915 are the gift from Dirk Landgraf, pSIM18 containing lambda RED function is available from one of our labs (See http://redrecombineering.ncifcrf.gov/strains--plasmids.html).

### Reagents

Phusion High-Fidelity DNA polymerase is from Biolabs. PCR product is purified with Qiagen PCR purification kit. L-(+)-Arabinose is from Calbiochem. Desalting oligos are ordered from Integrated DNA Technologies (IDT) [Table S2]

### Growth media

For standard culture of bacteria, cells were grown in liquid LB containing 1.0% (w/v) tryptone, 0.5% (w/v) yeast extract and 0.5% (w/v) NaCl. MOPS Rich Glycerol medium is a MOPS buffered media supplemented with ACUG, supplement EZ (amino acids and vitamins) as described by Neidhardt *et al*.^47^ For Mops glycerol we used 0.4% glycerol in the MOPs medium, and for Mops mannose, we used 0.2% mannose. Mops sorbital + 6aa and Mops glycerol + 6aa contains 5 ug/ml L-Methionine, L-Histidine, L-Arginine, L-Proline, L-Threonine, L-Tryptophan, respectively added to Mops sorbital and Mops glycerol medium. Mops Glucose rich medium had 0.2% glucose added to MOPS rich Glycerol medium; 0.1% arabinose was added for induction. For sucrose selection, NaCl was omitted and 6% (wt/vol) sucrose was added to LB. 12.5 ug/ml tetracycline, 10 ug/ml chloramphenicol, 75 ug/mL hygromycin and 50 ug/ml spectinomycin were used for selection.

### Growth rate measurement experiments

We used custom-made automated mini-turbidostats (TSTATs) to measure the growth rate of all strains described in the results during exponential growth phase of the cells (See the detail description in the Supplementary information).

### Test of reversibility of tCRISPRi

We diluted SJ_XTL174 10^6^-fold from cultures in LB broth to MOPS rich glycerol medium, removed 1 ml of cells at OD_600_ = 0.045 as the no induction control and then added arabinose at 0.1% to induce the dCas9 expression, and removed 1 ml samples from the T-STAT vial at different time points for 7 hours. After 7-hours induction, 10 ml of cells were concentrated by centrifugation, washed twice with Mops rich glycerol medium, and the pellet suspended in 20 ml MOPS Rich Glycerol without arabinose. These cells were placed in the T-STAT, and at different time points 1 ml of the culture was removed and fixed by formaldehyde, to allow cell size and fluorescent density measurements (Fig. 4d).

### DNA recombineering, selection & counter-selection and P1 transduction

The lambda Red recombineering was performed using lambda recombination functions provide by pSIM18 following published methods^48^,^49^, *tet*-*sacB* selection and counter-selection was described previously^28^, P1 transduction was carried out according to published methods^50^.

### Microscopy and image analysis

Images were acquired at 100× magnifications using Nikon Ti-E microscope equipped with a Neo sCMOS camera (Andor) and Nikon NIS-Elements software. For the cell length measurements, phase contrast technique was used, and images have 2560×2160 resolution and 16 bit grayscale. The fluorescent images for msfGFP and YFP cells were obtained with illumination by an OBIS 488 nm laser from Coherent and the 59022 filter cube from Chroma. For each cell, the fluorescence signal was integrated and normalized by the projected area of the cell after background subtraction. Illumination across the field of view was homogeneous with less than 5% variations. For each experimental condition, we acquired 150–200 images containing at least 10,000 cells. We developed and used custom high-throughput image analysis software optimized for our experiments using Python and OpenCV library. The length of filamentous cells for SJ_XTL229 (*ftsZ* CRISPRi) in Fig. 6c were measured manually using a contour of each cell because of their abnormal lengths, curved morphologies, and frequent intersections with each other. For [arabinose] = 0.025% and above, at least 300 filamentous cells were measured for each growth condition.

### Data analysis

**Figure 2:**
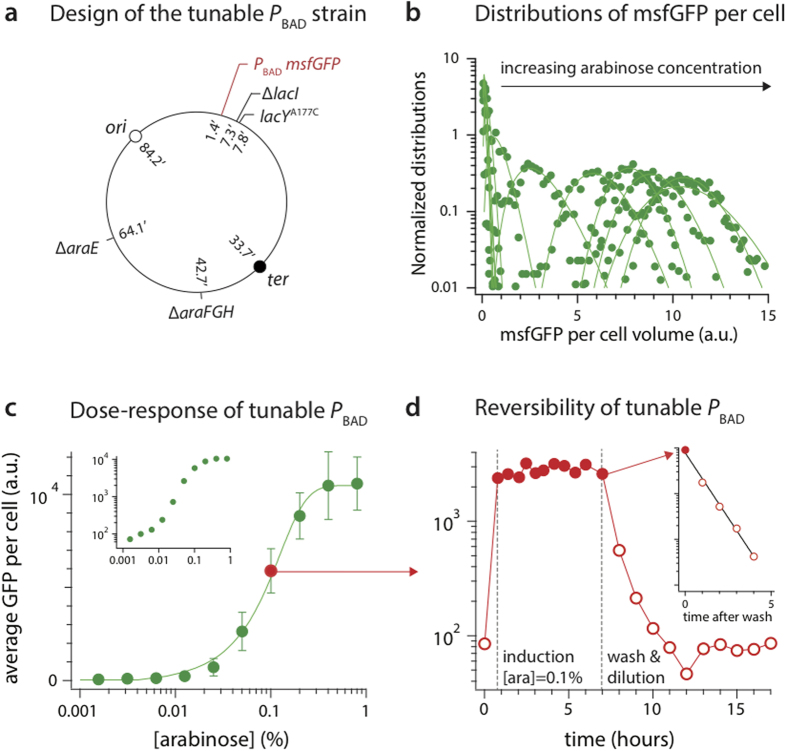
*P*_BAD_-*msfGFP*. (**a**) Design of *P*_BAD_-*msfGFP* strain. (**b**) Unlike a typical inducible promoter with a bimodal “on/off” switch-like behavior, expression by *P*_BAD_ in this modified strain shows a gamma distribution^27^,^30^ (filled circles = data, line = gamma distribution fit). Each gamma distribution represents an independent arabinose concentration trial. As the expression level becomes high, the distribution of msfGFP per cell volume becomes more symmetric and approaches Gaussian. The arabinose concentrations correspond to the ones used in (**c**). (**c**) The dynamic range of *P*_BAD_ in this strain is more than 100-fold (filled circles = data, line = dose-response sigmoid curve; error bars = standard deviations). (inset) Data in log-log scale. (**d**) Response of *P*_BAD_ is fast and reversible. (Inset) The first five data points from wash in semi-log scale (t = 0 is when arabinose is removed). The line is an exponential fit, demonstrating an immediate exponential dilution of msfGFP by growth upon removal of arabinose induction. Filled circle = No induction, open circle = induction at [arabinose] = 0.1%.

**Figure 3:**
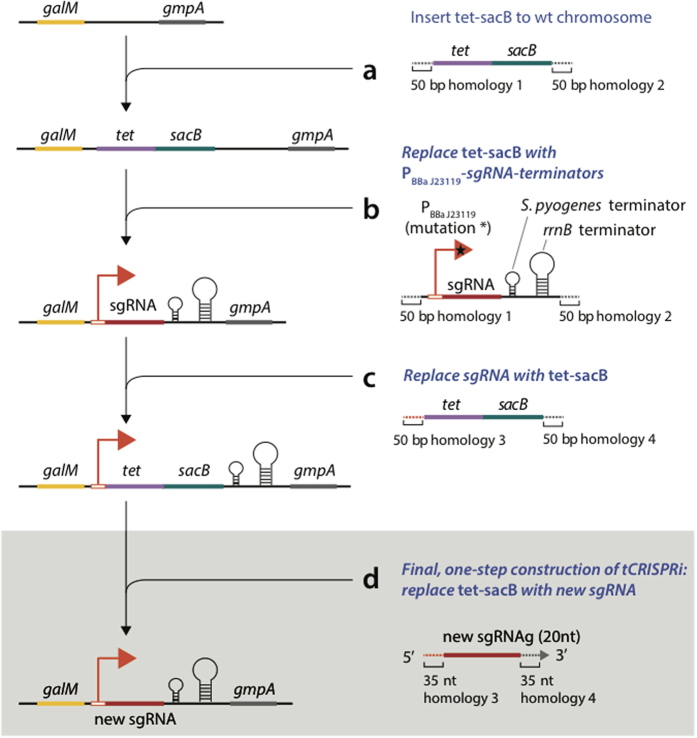
One-step construction of tCRISPRi. (**a**) Recombineering was used to introduce a PCR product encoding the selectable/counter-selectable genetic marker *tet-sacB*. The *tet*-*secB* is flanked by 50 bp homologies in the cassette between *galM* and *gmpA* in a strain containing the tunable tCRISPRi *P*_BAD_ system that expresses dCas9. (**b**) Using the same 50 bp homologies, the *tet-sacB* cassette was cleanly replaced by a PCR product derived from amplifying DNA from a plasmid encoding the synthetic promoter pBBaJ23119, an sgRNA targeting sequence, the Cas9 binding element, and two transcription terminators. (**c**) The *tet-sacB* cassette was used again to replace precisely just the sgRNA targeting sequence on the chromosome and to generate the generic strain that is used to insert new sgRNA cassettes, targeting any new DNA sequence. (**d**) In the final step (Shaded), recombineering is used to introduce a single oligonucleotide encoding the new sgRNA targeting sequence, flanked by 35 nt of homology to the sgRNA cassette, replacing *tet-sacB*. The total length of the oligo is 90 nt which has 35 nt homology on the 5′ end and 3′ end respectively, 20 nt sgRNA sequence in the middle. This final step is the only step the end user needs to execute to construct a strain for a new gene of interest.

**Figure 4:**
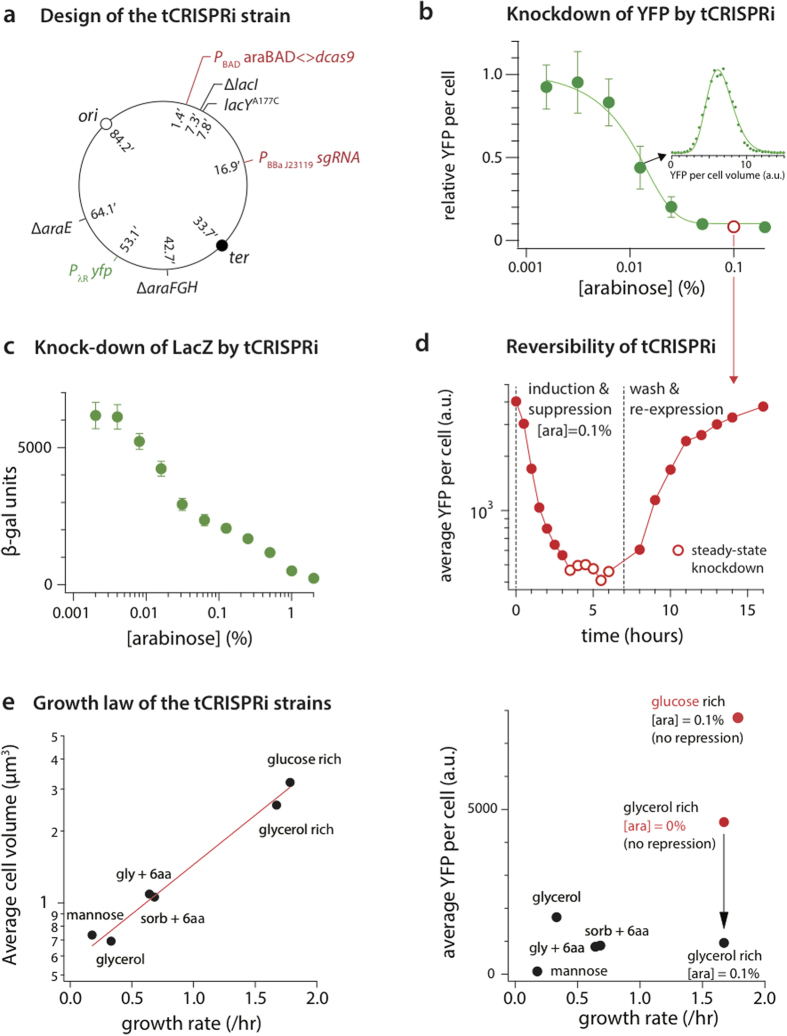
Tunable gene expression suppression by tCRISPRi. (**a**) Design of tCRISPRi. (**b**) tCRISPRi shows a 10-fold dynamic range of knockdown for yellow fluorescent protein (YFP) constitutively expressed under the strong phage lambda promoter (pR). (Inset) The expression of gene of interest (*yfp*) upon induction and suppression also shows a gamma distribution [circles = data, line = dose-response sigmoid curve]. (**c**) β-galactosidase assay also confirms repression of lacZ expression by tCRISPRi. (filled circles = data, line = dose-response sigmoid curve). (**d**) tCRISPRi is reversible. Shown here is knockdown and recovery of YFP expression at [arabinose] = 0.1% corresponding to the empty circle in (**b**) Once repression of *yfp* reaches steady state (indicated by a series of open circles), cells were washed and re-grown in a fresh medium without arabinose. The vertical line at t = 7 hours indicates the washing point (**e**) Robust physiological behavior of the tCRISPRi strains. The average cell size shows an exponential dependence on the nutrient-imposed growth rate^30^. YFP is repressed to a similar level at [arabinose] = 0.1%, with the exception of a catabolite repressor like glucose as a carbon source in the medium (see text).

The distribution of fluorescent proteins (msfGFP and YFP) were fitted using a gamma distribution function as suggested by by Taniguchi *et al*.^27^. The dose-response curves in Figs 2 and 3 were fitted using a standard sigmoid function: *f*(*x*) = *f*_max_/[1 + exp(*x* − *x*_1/2_)/γ], where *x* is the inducer concentration, γ is the induction rate constant, *f*_max_ is the max induction level, *x*_1/2_ is the half-max point to be fitted.

### β-Galactosidase Assay

Four independent colonies of SJ_XTL360 were grown overnight in LB broth at 30 °C. Cultures were diluted 1:1000 in fresh LB broth, then grown at 37 °C to OD_600_ = 0.03. Cultures were then diluted 1:100 to LB broth, and grown an additional hour until OD_600_ = 0.01. Each of the four independent cultures was divided into 12, 2 mL, cultures and arabinose was added to each to final concentrations of 2.0%, 1.0%, 0.5%, 0.25%, 0.125%, 0.0625%, 0.0313%, 0.0156%, 0.0078%, 0.0039%, 0.00195% and 0%. These cultures were grown 2.25 hours on a roller drum in a 37 °C incubator, to OD_600_ = 0.6. The cells were transferred to an ice-water bucket for 10 min. 50 uL of each culture was assayed for β-galactosidase activity^51^.

### RNA-Seq

Each of the 4 strains was pre-grown in LB media overnight at 37 °C followed by 3 successive 10^−3^ back-dilutions in MOPS Rich Glycerol at 37 °C. Turbidostats were inoculated with 1 mL each of the final back dilution batch cultures, then grown at 32 °C overnight. Turbidostat cultures were grown in MOPS Rich Glycerol at 37 °C for at least 2 back-dilutions from OD_600_ = 0.02 to 0.05 before collection. For each pair of induced/uninduced cultures, 5 mL of exponential culture was added directly from the turbidostat culture vial to a 15 mL conical tube on ice containing 0.65 mL of 5% w/v phenol in ethanol and mixed by inverting. Cells were subsequently pelleted in a clinical centrifuge for 30 minutes, after which the supernatant was aspirated and the pellet resuspended in 50 uL of 1× TMN followed by 1 mL of TRIzol Reagent. RNA from the lysed cells was prepared followed by DNase I treatment as the manufacturer’s instructions. The resulting DNA-free RNA was purified by phenol/chloroform/IAA extraction and ethanol precipitation. Libraries for sequencing were prepared using NuGEN Ovation Prokaryotic Complete RNA-Seq reagents with the exception of the substitution of *E. coli*-specific 16 S, 23 S, and 5 S rRNA depletion oligonucleotides in place of the multi-species oligonucleotide mix provided by the manufacturer. The libraries were subsequently pooled and sequenced with an Illumina MiSeq v3 150-cycle kit in single-read mode. Modified MG1655 genomes were constructed using Artemis (Broad Institute) and reads were subsequently aligned using Bowtie2.

### Fluorescent transcriptional reporter

**Figure 5:**
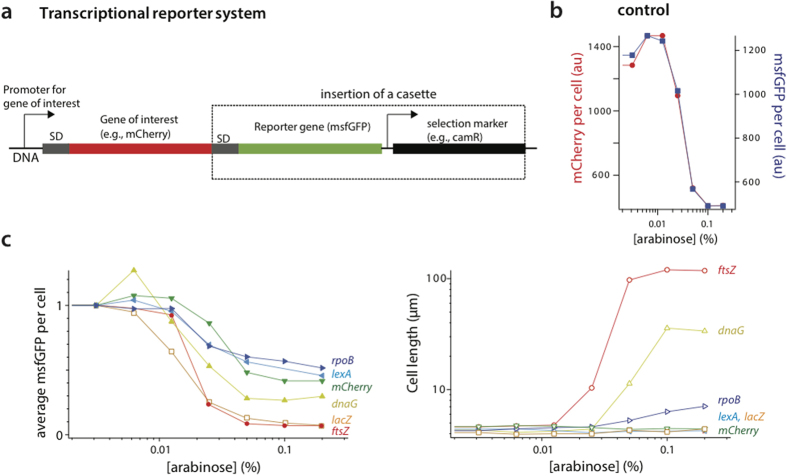
Application of tCRISPRi to essential and nonessential genes. (**a**) Design of the fluorescent transcriptional reporter. (**b**) Control of the transcriptional reporter (*msfGFP). mCherry* is the gene of interest, and its fluorescence level changes as the same rate as the transcriptional reporter (*msfGFP*) under knockdown of *mCherry* by tCRISPRi. (**c**) Knockdown of *ftsZ, dnaG, and rpoB* (essential genes) and *lacZ, lexA, mCherry* (nonessential genes). The expression level of all six genes decreased by tCRISPRi from their wildtype level up to 26 fold. Cell length of the three essential genes all increased by knockdown, whereas cell length remained unchanged for the nonessential genes.

**Figure 6:**
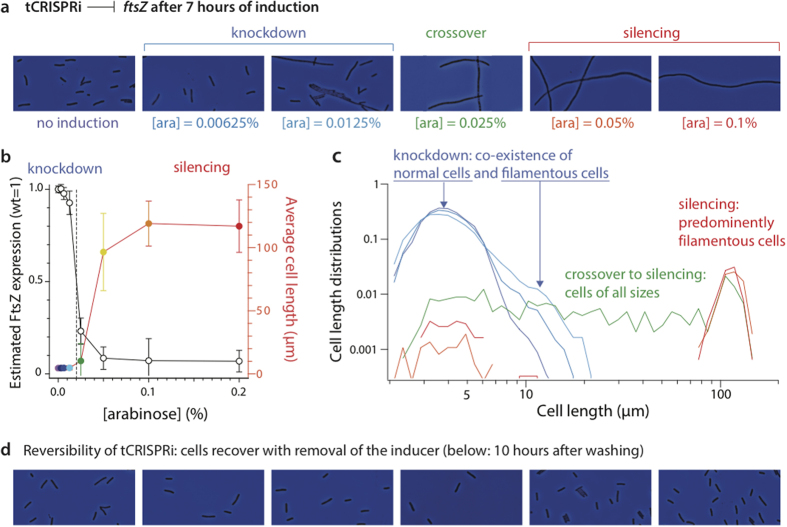
tCRISPRi on FtsZ reveals gene expression level dependent cell division regulation. Colors correspond to different arabinose concentrations as shown in (**a**). (**a**) Use of tCRISPRi to knockdown FtsZ expression to levels below the threshold required for cell division. At low repression of *ftsZ* most cells are normal, but a subpopulation of cells exclusively filament [see also cell size distributions in (**c**)]. As the suppression of FtsZ expression increases, all cells become filamentous and do not divide. (**b**) The threshold transition occurs when the level of FtsZ expression is approximately half the wild-type level. Cell sizes for [arabinose] > 0.05% are likely an underestimate, since many cells were larger than the field of view of the microscopy. The error bars indicate standard deviations of distributions. (**c**) Cell length distributions in log-log scale show crossover from knockdown (blue) to silencing (red) at [arabinose] = 0.0625% (green), with the cell population showing all lengths from 2 μm up to over 100 μm. (**d**) Reversibility of tCRISPRi is confirmed as all cells recover their normal size once the inducer is removed from the culture.

**Figure 7:**
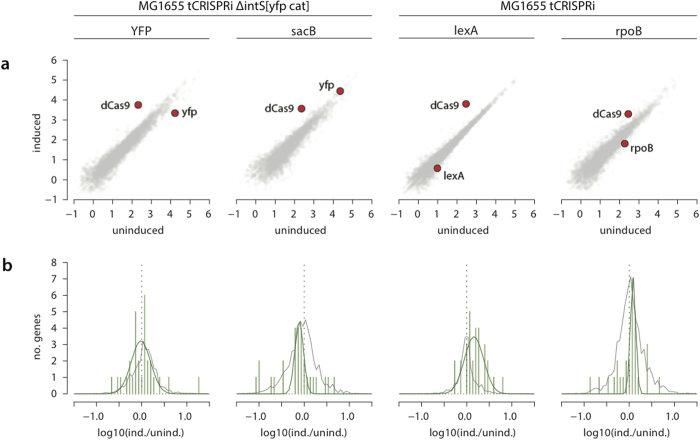
Off-target analysis by RNA-Seq. (**a**) log-log coplot of expression level (RPKM values) for all genes in both the uninduced (x axis) and induced (y axis) cultures. (**b**) distributions of LexA-regulated genes (green lines) compared to all genes (grey, proportionally rescaled). Individual gene changes are plotted in Supplementary Figure 6c.

We engineered a reporter gene, *msfGFP*, downstream of the tCRISPRi-targeted gene to form a synthetic operon so that the target gene and *msfGFP* gene are transcribed as one mRNA. The chloramphenicol gene was used as a selection marker of the recombineering (Fig. 5a).

To test the fluorescent transcriptional reporter, we co-expressed mCherry and msfGFP from the same promoter. The sgRNA was designed for targeting *mCherry*. We observed that the average mCherry and msfGFP per cells gradually decreased at the same rate by the tCRISPRi (Fig. 5b, Figure S5). The Shine-Dalgarno (SD)-*msfGFP*-cam transcription cassette was inserted downstream of the essential genes *rpoB, dnaG, ftsZ* and non-essential genes *lexA, lacZ* by recombineering, respectively.

### sgRNA sequences for whole genome of K-12 MG1655

To make this tunable tCRISPRi system facile for users, oligos were designed for every potential sgRNA sequence present in the *E. coli* genome (NCBI GenBank NC_000913.3) to quickly and easily order and make each sgRNA. Based on the size of the bacterial genome, each 20-mer required for an sgRNA is likely to be unique.

To establish counts for 10-, 15-, and 23-mers in the genome, jellyfish v2.2.4 was run in canonical mode. For each annotated Open reading frame (ORF) in the genome, we first confirmed the start and stop codons (NTG for start, [TAG, TGA, TAA] for stop). This screen resulted in 81 failed ORFs, including 2 genes annotated as essential by EcoCyc v19.5 (*yafF, infC*). For the failed ORFs, we used ORF annotations from EcoCyc which completed the remaining 2 genes annotated as essential. The remaining non-essential ORFs (Table S3) were not processed further. For each of the successfully screened ORFs, we tested each CC-prefixed subsequence of length 10, 15, and 23 for its abundance in the NC_000913.3 genome as well as the abundance of CT- or TC-prefixed off-target matches. We additionally tested for the presence of CC- and CT/TC-prefixed 23 mers which matched the test subsequence with 1 or 2 mismatches outside of the PAM. The candidate sgRNA sequences were sorted by increasing quantity of region I matches and those with any exact, off-by-1, or off-by-2 matches were removed. In selecting sgRNAs, a positional limit of 150 nt from the start codon was sufficient to cover the majority of the genome. The remaining sgRNAs and the least abundant region I sequence for each gene is listed (Table S4). We provide these sequences as a starting point for those interested in construction of a high-throughput library or customized sgRNA construction.

## Additional Information

**How to cite this article**: Li, X.-t *et al*. tCRISPRi: tunable and reversible, one-step control of gene expression. *Sci. Rep.* **6**, 39076; doi: 10.1038/srep39076 (2016).

**Publisher's note:** Springer Nature remains neutral with regard to jurisdictional claims in published maps and institutional affiliations.

## Supplementary Material

Supplementary Information

Supplementary Dataset 1

## Figures and Tables

**Table 1:** Comparison of different CRISPRi system.

	Ref. 12	Ref. 21	This study
Organism	*E. coli*	*B. subtilis*	*E. coli*
gRNA	PCR, plasmid cloning, Gibson assembly	Reverse PCR, plasmid cloning, “integrating plasmids”	Oligo recombineering
	Strain construction requires multiple steps	Strain construction requires multiple steps	Strain construction requires single step
	Drug marker in the final construct	Drug marker in the final construct	No drug marker in the final construct
	Constitutive promoter from plasmids	Constitutive promoter from chromosome	Constitutive promoter from chromosome
dCas9 expression	Constitutive promoter from plasmids	xylose-inducible promoter from chromosome	*P*_BAD_ from chromosome
Leakiness	Information not available	~33% leaky	~7.5% leaky
Suppression level	*gfp* promoter sgRNA: 100X ORF sgRNA: 6×–35×	*rfp* ORF sgRNA: 150× Essential genes ORF sgRNA: >3×	*yfp* ORF sgRNA: >10× Other genes ORF sgRNA: 2×–32×
Genes tested	2	289	7

## References

[b1] Barrangou R. . CRISPR provides acquired resistance against viruses in prokaryotes. Science 315, 1709–1712 (2007). 17379808 10.1126/science.1138140

[b2] Garneau J. E. . The CRISPR/Cas bacterial immune system cleaves bacteriophage and plasmid DNA. Nature 468, 67–71 (2010). 21048762 10.1038/nature09523

[b3] Jinek M. . A programmable dual-RNA-guided DNA endonuclease in adaptive bacterial immunity. Science 337, 816–821 (2012). 22745249 10.1126/science.1225829 PMC6286148

[b4] Gasiunas G. . Cas9-crRNA ribonucleoprotein complex mediates specific DNA cleavage for adaptive immunity in bacteria. Proc. Natl. Acad. Sci. USA 109, E2579–86 (2012). 22949671 10.1073/pnas.1208507109 PMC3465414

[b5] Makarova K. S. . Evolution and classification of the CRISPR-Cas systems. Nat. Rev. Microbiol. 9, 467–477 (2011). 21552286 10.1038/nrmicro2577 PMC3380444

[b6] Mojica F. J. M. . Short motif sequences determine the targets of the prokaryotic CRISPR defence system. Microbiology 155, 733–740 (2009). 19246744 10.1099/mic.0.023960-0

[b7] Sapranauskas R. . The *Streptococcus thermophilus* CRISPR/Cas system provides immunity in *Escherichia coli*. Nucleic Acids Res. 39, 9275–9282 (2011). 21813460 10.1093/nar/gkr606 PMC3241640

[b8] Peters J. M. . Bacterial CRISPR: accomplishments and prospects. Curr. Opin. Microbiol. 27, 121–126 (2015). 26363124 10.1016/j.mib.2015.08.007 PMC4659716

[b9] Sternberg S. H. & Doudna J. A. Expanding the Biologist’s Toolkit with CRISPR-Cas9. Mol. Cell 58, 568–574 (2015). 26000842 10.1016/j.molcel.2015.02.032

[b10] Makarova K. S. . An updated evolutionary classification of CRISPR–Cas systems. Nat. Rev. Microbiol. 13, 722–736 (2015). 26411297 10.1038/nrmicro3569 PMC5426118

[b11] Qi L. S. . Repurposing CRISPR as an RNA-guided platform for sequence-specific control of gene expression. Cell 152, 1173–1183 (2013). 23452860 10.1016/j.cell.2013.02.022 PMC3664290

[b12] Bikard D. . Programmable repression and activation of bacterial gene expression using an engineered CRISPR-Cas system. Nucleic Acids Res. 41, 7429–7437 (2013). 23761437 10.1093/nar/gkt520 PMC3753641

[b13] Zalatan J. G. . Engineering complex synthetic transcriptional programs with CRISPR RNA scaffolds. Cell 160, 339–350 (2015). 25533786 10.1016/j.cell.2014.11.052 PMC4297522

[b14] Ji W. . Specific gene repression by CRISPRi system transferred through bacterial conjugation. ACS Synth. Biol. 3, 929–931 (2014). 25409531 10.1021/sb500036q PMC4277763

[b15] Jiang W. . Successful transient expression of Cas9 and single guide RNA genes in *Chlamydomonas reinhardtii*. Eukaryot. Cell 13, 1465–1469 (2014). 25239977 10.1128/EC.00213-14 PMC4248704

[b16] Jacobs J. Z., Ciccaglione K. M., Tournier V. & Zaratiegui M. Implementation of the CRISPR-Cas9 system in fission yeast. Nat. Commun. 5, 5344 (2014). 25352017 10.1038/ncomms6344 PMC4215166

[b17] Pyne M. E., Bruder M. R., Moo-Young M., Chung D. A. & Chou C. P. Harnessing heterologous and endogenous CRISPR-Cas machineries for efficient markerless genome editing in *Clostridium*. Sci. Rep. 6, 25666 (2016). 27157668 10.1038/srep25666 PMC4860712

[b18] Sebo Z. L., Lee H. B., Peng Y. & Guo Y. A simplified and efficient germline-specific CRISPR/Cas9 system for *Drosophila* genomic engineering. Fly 8, 52–57 (2014). 24141137 10.4161/fly.26828 PMC3974895

[b19] Morgan-Kiss R. M., Wadler C. & Cronan J. E. Jr. Long-term and homogeneous regulation of the *Escherichia coli araBAD* promoter by use of a lactose transporter of relaxed specificity. Proc. Natl. Acad. Sci. USA 99, 7373–7377 (2002). 12032290 10.1073/pnas.122227599 PMC124238

[b20] Siegele D. A. & Hu J. C. Gene expression from plasmids containing the *araBAD* promoter at subsaturating inducer concentrations represents mixed populations. Proceedings of the National Academy of Sciences 94, 8168–8172 (1997). 10.1073/pnas.94.15.8168 PMC21575 9223333

[b21] Peters J. M. . A Comprehensive, CRISPR-based Functional Analysis of Essential Genes in Bacteria. Cell 165, 1493–1506 (2016). 27238023 10.1016/j.cell.2016.05.003 PMC4894308

[b22] Diaz Ricci J. C. & Hernández M. E. Plasmid Effects on *Escherichia coli* Metabolism. Crit. Rev. Biotechnol. 20, 79–108 (2000). 10890453 10.1080/07388550008984167

[b23] Tal S. & Paulsson J. Evaluating quantitative methods for measuring plasmid copy numbers in single cells. Plasmid 67, 167–173 (2012). 22305922 10.1016/j.plasmid.2012.01.004 PMC3709585

[b24] Novick A. & Weiner M. Enzyme Induction as an all-or-None Phenomenon. Proc. Natl. Acad. Sci. USA 43, 553–566 (1957). 16590055 10.1073/pnas.43.7.553 PMC528498

[b25] Ochab-Marcinek A. & Tabaka M. Bimodal gene expression in noncooperative regulatory systems. Proceedings of the National Academy of Sciences 107, 22096–22101 (2010). 10.1073/pnas.1008965107 PMC3009792 21135209

[b26] Michel D. Kinetic approaches to lactose operon induction and bimodality. J. Theor. Biol. 325, 62–75 (2013). 23454080 10.1016/j.jtbi.2013.02.005

[b27] Taniguchi Y. . Quantifying *E. coli* proteome and transcriptome with single-molecule sensitivity in single cells. Science 329, 533–538 (2010). 20671182 10.1126/science.1188308 PMC2922915

[b28] Li X.-T., Thomason L. C., Sawitzke J. A., Costantino N. & Court D. L. Positive and negative selection using the *tetA*-*sacB* cassette: recombineering and P1 transduction in *Escherichia coli*. Nucleic Acids Res. 41, e204 (2013). 24203710 10.1093/nar/gkt1075 PMC3905872

[b29] Larson M. H. . CRISPR interference (CRISPRi) for sequence-specific control of gene expression. Nat. Protoc. 8, 2180–2196 (2013). 24136345 10.1038/nprot.2013.132 PMC3922765

[b30] Schaechter M., Maaloe O. & Kjeldgaard N. O. Dependency on medium and temperature of cell size and chemical composition during balanced grown of *Salmonella typhimurium*. J. Gen. Microbiol. 19, 592–606 (1958). 13611202 10.1099/00221287-19-3-592

[b31] Guzman L. M., Belin D., Carson M. J. & Beckwith J. Tight regulation, modulation, and high-level expression by vectors containing the arabinose P_BAD_ promoter. J. Bacteriol. 177, 4121–4130 (1995). 7608087 10.1128/jb.177.14.4121-4130.1995 PMC177145

[b32] Kuhlman T., Zhang Z., Saier M. H. Jr & Hwa T. Combinatorial transcriptional control of the lactose operon of *Escherichia coli*. Proc. Natl. Acad. Sci. USA 104, 6043–6048 (2007). 17376875 10.1073/pnas.0606717104 PMC1851613

[b33] Abudayyeh O. O. . C2c2 is a single-component programmable RNA-guided RNA-targeting CRISPR effector. Science 353, aaf5573–aaf5573 (2016). 27256883 10.1126/science.aaf5573 PMC5127784

[b34] Bi E. & Lutkenhaus J. Assembly Dynamics of the Bacterial MinCDE System and Spatial Regulation of the Z Ring. Annu. Rev. Biochem. 76, 539–562 (2007). 17328675 10.1146/annurev.biochem.75.103004.142652

[b35] Bi J. & Lutkenhaus E. FtsZ ring structure associated with division in *Escherichia coli*. Nature 354, 161–164 (1991). 1944597 10.1038/354161a0

[b36] Haeusser D. P. & William M. Splitsville: structural and functional insights into the dynamic bacterial Z ring. Nat. Rev. Microbiol. 14, 305–319 (2016). 27040757 10.1038/nrmicro.2016.26 PMC5290750

[b37] Buske P. J., Mittal A., Pappu R. V. & Levin P. A. An intrinsically disordered linker plays a critical role in bacterial cell division. Semin. Cell Dev. Biol. 37, 3–10 (2015). 25305578 10.1016/j.semcdb.2014.09.017 PMC4339304

[b38] Hill N. S., Kadoya R., K. C. D. & Levin P. A. Cell size and the initiation of DNA replication in bacteria. PLoS Genet. 8, e1002549 (2012). 22396664 10.1371/journal.pgen.1002549 PMC3291569

[b39] Tétart F., Albigot R., Confer A. & Jean-Pierre M. E. B. Involvement of FtsZ in coupling of nucleoid separation with septation. Mol. Microbiol. 6, 621–627 (1992). 1552861 10.1111/j.1365-2958.1992.tb01509.x

[b40] Mukherjee A., Saez C. & Lutkenhaus J. Assembly of an FtsZ Mutant Deficient in GTPase Activity Has Implications for FtsZ Assembly and the Role of the Z Ring in Cell Division. J. Bacteriol. 183, 7190–7197 (2001). 11717278 10.1128/JB.183.24.7190-7197.2001 PMC95568

[b41] Margolin W. Themes and variations in prokaryotic cell division. FEMS Microbiol. Rev. 24, 531–548 (2000). 10978550 10.1111/j.1574-6976.2000.tb00554.x

[b42] Tsai S. Q. . GUIDE-seq enables genome-wide profiling of off-target cleavage by CRISPR-Cas nucleases. Nat. Biotechnol. 33, 187–197 (2014). 25513782 10.1038/nbt.3117 PMC4320685

[b43] Baba T. . Construction of *Escherichia coli* K-12 in-frame, single-gene knockout mutants: the Keio collection. Mol. Syst. Biol. 2, 2006.0008 (2006). 10.1038/msb4100050 PMC1681482 16738554

[b44] Du D., Dan D. & Qi L. S. CRISPR Technology for Genome Activation and Repression in Mammalian Cells. Cold Spring Harb. Protoc. 2016, db.prot090175 (2016). 10.1101/pdb.prot090175 26729910

[b45] Wang H. H. . Programming cells by multiplex genome engineering and accelerated evolution. Nature 460, 894–898 (2009). 19633652 10.1038/nature08187 PMC4590770

[b46] La Russa M. F. & Qi L. S. The New State of the Art: Cas9 for Gene Activation and Repression. Mol. Cell. Biol. 35, 3800–3809 (2015). 26370509 10.1128/MCB.00512-15 PMC4609748

[b47] Neidhardt F. C., Bloch P. L. & Smith D. F. Culture medium for enterobacteria. J. Bacteriol. 119, 736–747 (1974). 4604283 10.1128/jb.119.3.736-747.1974 PMC245675

[b48] Sawitzke J. A. . In Methods in Enzymology 79–102 (2013). 10.1016/B978-0-12-420067-8.00007-6 PMC7511988 24182919

[b49] Thomason L. . In Current Protocols in Molecular Biology 106, 1.16.1–1.16.39 (2014). 24733238 10.1002/0471142727.mb0116s106

[b50] Thomason L. . In Current Protocols in Molecular Biology 1.17.1–1.17.8 (2007). 18265391 10.1002/0471142727.mb0117s79

[b51] Miller J. H. In Experiments in molecular genetics 352–360 (Cold Spring Harbor Laboratory Press, 1972).

