# Supplementary material for: tCRISPRi: tunable and reversible, one-step control of gene expression
Source: Sci Rep. 2016 Dec 20;6:39076. doi: 10.1038/srep39076 (PMC5171832; doi:10.1038/srep39076)
Supplement: Supplementary Information [file srep39076-s1.pdf]

Supplementary information:

tCRISPRi: tunable and reversible, one-step control of gene expression

Xin-tian Li[1*,†], Yonggun Jun[1,‡], Michael J. Erickstad[1], Steven D. Brown[1], Adam Parks[2], Donald L. Court[2] & Suckjoon Jun[1]**

[1] Section of Molecular Biology, Division of Biological Sciences and Department of Physics, UC San Diego, La Jolla, CA 92093, USA

[2] Gene Regulation and Chromosome Biology Laboratory, Center for Cancer Research, National Cancer Institute at Frederick, Frederick, Maryland 21702, USA

[†]Current address: Basic Science Program, GRCBL-Molecular Control & Genetics Section, Frederick National Laboratory for Cancer Research, Leidos Biomedical Research, Inc., Frederick, MD 21702, USA

[‡]Current address: Department of Physics, National Central University, Taiwan

*** Email: xintianli@gmail.com**
** Email: suckjoon.jun@gmail.com

Growth rate measurement experiments

We used custom-made automated mini-turbidostats (TSTATs) to measure the growth rate of all strains in the results during balanced growth. The cell cultures in the TSTATs were not held at constant turbidity. Instead, the TSTATs were set to allow the accumulation of turbidity up to a set threshold. Upon reaching this upper turbidity threshold each TSTAT diluted its culture until a defined lower turbidity threshold was achieved. After dilution, the culture in each TSTAT was then allowed to grow freely again until it again reached the upper turbidity threshold. This process of repeated growth and dilution was used to maintain the cultures in a state of balanced growth. The turbidity of the cellular suspensions was measured at 15-second intervals. With the TSTAT operating in this fashion, the time series of turbidity measurements for a suspension of cells doubling at a constant rate (an uninhibited wild-type strain) naturally resulted in a train of

exponential saw teeth. The individual exponential curves in each saw tooth were fit in order to measure the doubling times. Each TSTAT has its own fixed wavelength spectrophotometer for taking real-time turbidity measurements. The spectrophotometer componentry of each TSTAT was built into a culture vial holder made from 1.625” diameter Delrin round stock with a vertical bore (1”) and 2 interior o-ring grooves (dash number 213) ideal for holding 8 dram (32 ml) glass vials (~0.2” from each end of its ~1.5” height). Each holder has a horizontal bore (0.191”) through its diameter (0.550” from bottom), with one 600 nm T-1¾ form factor LED with a 15° viewing angle press-fitted into one end such that it faces opposite to a 570 nm (FWHM ~350 nm) broad band T-1¾ form factor phototransistor press fitted into the other end. With a culture vial placed in the vertical bore of the holder the LED and phototransistor act as a simple fixed wavelength spectrophotometer. An Arduino Mega 2560 microcontroller board powers the LEDs for each TSTAT via its PWM outputs and measures the current through the LEDs and the phototransistors of each TSTAT by taking voltage measurements across shunts in series with the components via its ADC’s. In each 15 second measurement cycle, the Arduino activates its spectrophotometer (waits 2 seconds for it to warm up) and quickly (~1s) takes a series of 100 measurements, after which it powers down the spectrophotometer and reports the average value along with the epoch time and other status information to a laptop computer via serial connection. The Arduino Mega 2560 microcontroller board is programmed for autonomous operation in the event of loss of computer connection. The Arduino Mega 2560 microcontroller board also streams a duplicate log of all serial output to an Arduino Uno microcontroller board with an SD card shield acting as an auxiliary logger. A Java GUI on the laptop provides the main control and logging functionality including the ability to get and set a blank value for each spectrophotometer. Separate pneumatically driven media bottles supply each TSTAT with

growth media for dilution. The flow in the dilution/media lines is mechanically controlled by pinch valves (normally closed Bio-Chem). A regulated diaphragm pump supplies driving pressure (15 kPa) to the headspace of each media bottle as well as the compressed air flow which bubbles the cultures in each TSTAT vial. Small inner diameter (0.010”) peek tubing segments (1” length) are used as capillary gas resistors to ensure even delivery of bubbling to each vial. A custom machined plug assembly threads onto each culture vial sealing it airtight with a dash number 014 o-ring. Each plug has 4 stainless steel stems (19 ga.) the shortest and longest of which are the media-in and air-in lines respectively. The two intermediate length stems function as output lines the lower (longer) of which is used during dilution to establish the fill level of the vial. A “wye” pinch valve (Bio-Chem) controlled by the microcontroller ensures that only one of the two output lines has flow at any given time. A small (4X12 mm) stir bar provides additional agitation to the culture.

Strain construction

Construction of a strain for tunable expression of P_{BAD}. We used two main techniques to construct a plasmid-free dose-inducible gene expression system: DNA recombineering and *tet-sacB* selection/counter-selection. The parent strain is low motility MG1655 (Table S1). The Red DNA recombineering plasmid pSIM18 was transformed to the low motile MG1655. The arabinose transporters *araE* and the lactose inhibitor gene *lacI* were seamlessly deleted. SJ_XTL166 was made by introducing a the lactose transporter gene mutation *lacY* A177C, replacing the arabinose transporter gene *araFGH* by a spectinomycin resistant gene. The *araD<>tet-sacB-amp* was transferred from XTL298 (Table S1) to SJ_XTL166 by P1 transduction to make SJ_XTL175. The monomeric super-folder *gfp* (*msfGFP*) gene was

amplified from the plasmid pDHL1029 by PCR to create a start codon at the N-terminus for *msfGFP* and an optimized Shine-Dalgarno of the P_{BAD} promoter. Finally, SJ_XTL177 was made by replacing the *tet-sacB-amp* in SJ_XTL175 with *msfGFP* to create P_{BAD}-*msfGFP*, which is the fluorescent reporter construct for the P_{BAD} promoter.

To create wobble mutations in the sgRNA targeted region of *yfp*, we added pSIM18 to strain SJ_XTL174 and then inserted the *tet-sacB* cassette into the *yfp* sgRNA region on the chromosome. Next the *tet-sacB* cassette was replaced by recombineering with oligos of SJ_XT339, SJ_XT340, SJ_XT341, SJ_XT342 and SJ_XT343, respectively (Figure S6), to create the five YFP mutant strains SJ_XTL557, SJ_XTL558, SJ_XTL559, SJ_XTL560 and SJ_XTL561. YFP sgRNAs inhibition of YFP activity for each strain was then measured as a function of increasing the number of mismatched bases with the same sgRNA.

dcas9/sgRNA construction in the *E. coli* chromosome. The *dcas9* gene was amplified from the pdcas9 plasmid. The strain SJ_XTL212 (P_{BAD}-*dcas9*) was made by replacing the *tet-sacB-amp* in the strain SJ_XTL175 with *dcas9* by DNA recombineering (see Figures S8 and S9).

We inserted a sgRNA sequence to the chromosome in the following procedure: Starting from the strain XTL333 containing the *tet-sacB* selection/counter-selection cassette inserted between *galK* and *gpmA* genes (Table S1, Figure 3, Figure S1). Replace the *tet-sacB* with PCR cassette, $P_{\mathrm{BBa\text{-}J23119}}$-*sgRNA*-(*S. pyogenes terminato*r)-(*rrnB terminator*), which was amplified from the plasmid PgRNA-bacterial (Figure 3). Replace the sgRNA fragment with *tet-sacB* again by recombineering to make the strain SJ_XTL123. P1 transduce the region ($P_{\mathrm{BBa\text{-}J23119}}$-*tet-sacB*-

handle-(*S. pyogenes terminator*)-(*rrnB terminator*) in SJ_XTL123 to SJ_XTL212 (P_{BAD}-*dcas9*) to make the strain SJ_XTL219. The only step to make customized sgRNA is to design an 90 nt oligo with 35 nt homologies at both ends, and 20 nt sgRNA DNA sequence in the middle (Figure S1), make sure that there is a PAM site (NGG)[50] next to the sgRNA in the target region in the chromosome, BAC, phage or plasmid. Copy the 90 nt sequence which is the same direction with *galM* coding sequence to design the lagging strand recombineering oligo (Figure S1). The modified pBBa-J23119 promoter in the plasmid PgRNA-bacterial was converted to the wild type pBBa-J23119 promoter by designing the homology containing the wild-type pBBa-J23119 promoter sequences in this step (Figure S1). SJ_XTL174 is the fluorescence reporter strain to test tCRISPRi by measuring the YFP fluorescence per cell.

Transcription reporter construction. A fragment of *cm-msfGFP*-SD-*mcherry*-SD-pBBa_J23119 was inserted between *sgcX* and *yjhP* in the *E.coli* chromosome by multiple recombineering, The *mcherry* was amplified from the plasmid pDHL915 by PCR. Then the fragment *cm-msfGFP*-SD was amplified by PCR and inserted immediately after the stop codon, selecting with chloramphenicol. The sequence of SJ_XTL410 (mcherry reporter strain) is shown in the Figure S5.

Induction of cells containing tCRISPRi

Single colonies are picked and inoculated to 3 ml LB broth, and grown overnight at 37 ^{o}C. A 1:1000 dilution is made to the MOPS rich glycerol medium and grown at 37°C to OD600=0.3, and another 1:100 dilution is made to MOPS rich glycerol medium containing different arabinose concentrations and grown at 37°C for 4 hours. The cells are taken from the culture or STAT vials and transferred to a 2% agarose pad made with the MOPS rich glycerol medium.

Pictures are taken under the microscope to measure cell size and fluorescent density.

Fluorescence measurement from P_{BAD} in the tCRISPRi system. The strain SJ_XTL177 (containing P_{BAD}-*msfGFP*) batch culture was induced as described in the Materials and Methods. The working arabinose concentrations were 1.6%, 0.8%, 0.4%, 0.2%, 0.1%, 0.05%, 0.025%, 0.0125%, 0.00625%, 0.00313%, 0.00156% and 0% for all experiments unless specified otherwise. The duration of induction of P_{BAD} was 4 hours at 37C, after which image acquisition started immediately.

Establishment of a transcription reporter system to monitor gene specific tCRISPRi inhibition.

In a traditional transcription reporter, the regulatory region from a gene of interest is cloned on a plasmid upstream of the reporter gene [51]. However, to decide the DNA length that includes the promoter and the regulatory region is determined on a case by case basis and even then may not include all of that gene's regulatory region. This would certainly be the case if the some of the regulatory region is located inside the coding sequence of the gene of interest. Moreover, by using a multi-copy plasmid, the regulatory region could compete with (titrate) regulatory factors for the gene of interest.

A second traditional method is to clone a reporter gene so that it is translationally fused with a gene of interest[52]. But the protein fusion generated could affect activity of the protein of interest as well as the reporter activity as well. Both traditional methods generally use plasmids, which could affect the physiology of the cells.

The advantages of our reporter system are: 1) the transcription level of the gene of interest can be monitored without disturbing that gene's regulatory components. 2) Because the reporter is cloned on the chromosome downstream of the gene of interest, it leaves the entire regulatory region of that gene intact so as to reflect in the reporter the specific gene's normal transcription level. 3) it is a plasmid-free system. 4) Unlike a fusion reporter, there is no folding or other protein fusion problem of the gene product of interest and the reporter protein.

Table S1. Strains used

Strain name	Genotype	Note
MG1655	F- λ- *rph*-1	(Low motile)[53]
XTL298	*W3110* Δ*lacU*169 *gal*490 *pgl*Δ*8*[λ*cI*857 Δ(*cro-bioA*)] *araD*<>*tetA*-*sacB*-*amp*	[28]
XTL333	*MG1655 galM*<*tet-sacB*>*gmpA* pSIM18	[28]
SJ_XTL123	*MG1655 galM*<pBBa-J23119-*tet*-*sacB*-handle-(S. pyogenes terminator)-(*rrnB* terminator)>*gmpA* pSIM18	This study
SJ_XTL166	*MG1655 lacY* A177C, *spec*<>*araFGH*, Δ*lacI* Δ*araE* pSIM18	P_{BAD}
SJ_XTL174	SJ_XTL219 *yfp* sgRNA, *intC*::[P(λR):*yfp*	CRISPRi reporter P_{BAD}-*dcas9*, *yfp* sgRNA
SJ_XTL175	SJ_XTL166 *araD*<>*tet-sacB-amp*	This study
SJ_XTL177	SJ_XTL175 *araBAD*<>*msfGFP* pSIM18	P_{BAD} *msfgfp* reporter
SJ_XTL212	SJ_XTL175 ara*BAD*<>*dcas9* pSIM18	P_{BAD}-*dcas9*
SJ_XTL219	SJ_XTL212 *galM*<pBBa-J23119-*tet*-*sacB*-handle-(S.pyogenes terminator)-(*rrnB* terminator)>*gmpA* pSIM18	Mother strain to make P_{BAD}-dcas9/sgRNA
SJ_XTL228	SJ_XTL219 *lexA* sgRNA	This study
SJ_XTL229	SJ_XTL219 *ftsZ* sgRNA	This study
SJ_XTL323	SJ_XTL219 no sgRNA	This study
SJ_XTL320	SJ_XTL219 *rpoB* sgRNA	This study
SJ_XTL321	SJ_XTL219 *dnaG* sgRNA	This study
SJ_XTL360	SJ_XTL219 *lacZ* sgRNA	This study
SJ_XTL404	MG1655 *sgcX*<*cm-msfGFP-SD-attB*-pBBa_J23119>*yjhP*	This study
SJ_XTL408	SJ_XTL229 *ftsZ*<>*ftsZ*-SD-*msfGFP-cm*	*ftsZ* transcription reporter
SJ_XTL410	SJ_XTL219 *mcherry* sgRNA, *glvC*<*kan-mcherry-SD-msfGFP-cm*>*yidP*	*mcherry* transcription reporter
SJ_XTL427	SJ_XTL174 *araD*< >*tet-sacB*	leak measurement
SJ_XTL454	SJ_XTL426 *sacB* sgRNA *intC*::[P(λR):*yfp*]	This study
SJ_XTL457	SJ_XTL228 *lexA*<>*lexA*-SD-*msfGFP-cm*	*lexA* transcription reporter
SJ_XTL458	SJ_XTL320 *rpoB*<>*rpoB*-SD-*msfGFP-cm*	*rpoB* transcription reporter
SJ_XTL459	SJ_XTL321 *dnaG*<>*dnaG*-SD-*msfGFP-cm*	*dnaG* transcription reporter
SJ_XTL460	SJ_XTL360 *lacZ*<>*lacZ*-SD-*msfGFP-cm*	*lacZ* transcription reporter
SJ_XTL557	SJ_XTL174 *yfp* mutation_1	This study Figure 6S
SJ_XTL558	SJ_XTL174 *yfp* mutation_2	This study Figure 6S
SJ_XTL559	SJ_XTL174 *yfp* mutation_3	This study Figure 6S
SJ_XTL560	SJ_XTL174 *yfp* mutation_4	This study Figure 6S
SJ_XTL561	SJ_XTL174 *yfp* mutation_5	This study Figure 6S

Table S2 Oligos used

Oligo name	Sequence	Notes
SJ_XT4	AACAGCACTGGGCGCCGACC	check *tet-sacB* insertion
SJ_XT33	AAGTGTCTATAATCACGGCAGA	check P_{BAD}*dCas9*
SJ_XT44	TGTTCACCATCAATAATCAGTTTGTTTTCTGGCTGGGCTCTGGCTGTGCATCCTAATTTTTGTTGACACTCTATC	make *lacY<>tet-sacB* forward
SJ_XT45	AAGAGGGCGCATCCGTTTTGGCGAAAAAGAGTAAAACGGCGAGGATGAGATCAAAGGGAAAACTGTCCATATGC	make *lacY<>tet-sacB* reverse
SJ_XT47	AGTAATTTCGAATTTGGTCGCGC	check *lacY* A177C
SJ_XT48	GATATGTTGGTCGGATAAGGCG	check l*acY* A177C
SJ_XT51	CCATCACATAACGACATGTCG	check *araE<>tet*
SJ_XT52	TGTCATTCGTTTTTGCCCTACACAAAACGACACTAAAGCTGGAGAGAACCACCGTGGAAACGGATGAAGGC	make *araFGH<>spec* forward
SJ_XT53	GGTTGGCTATGGTGGAAAAAACGCTAAATTGTTGCAGAAAAAAGCATCAAGGGCTTATTATGCACGCTTAA	make *araFGH<>spec* reverse
SJ_XT54	GCTAAAACTATGTCAACACAGTCA	check *araFGH<>spec*
SJ_XT55	CTGCTGTGGATTATTACCGG	PCR l*acY* A177C
SJ_XT56	CGCAAAGAACATAATCGAGGC	PCR *lacY* A177C
SJ_XT110	CGCTTTTTATCGCAACTCTCTACTGTTTCTCCATACCCGTTTTTTTGGATAGGAGGATGAAACGATGGATAAGAAATACTCAATAGGC	make P_{BAD}*dcas9* forward
SJ_XT111	TGAgTATAGCCTGGTTTCGTTTGATTGGCTGTGGTTTTATACAGTCATTAGTCACCTCCTAGCTGACTCA	make P_{BAD}*dcas9* reverse
SJ_XT112	CCCGTTGTCCGTCGCCAGCCAG	check P_{BAD}*dcas9*
SJ_XT113	TGAACGTCATCCTATTTTTGGA	Sequencing P_{BAD}*dcas9*
SJ_XT114	GCTGATTTGTTTTTGGCAGCTAAG	Sequencing P_{BAD}*dcas9*
SJ_XT115	GCATGGATGACTCGGAAGTC	Sequencing P_{BAD}*dcas9*
SJ_XT116	ACATATGCTCACCTCTTTGATG	Sequencing P_{BAD}*dcas9*
SJ_XT117	TCTCCAAAATGGAAGAGACATG	Sequencing P_{BAD}*dcas9*
SJ_XT118	AATGCCGTCGTTGGAACTGC	Sequencing P_{BAD}*dcas9*
SJ_XT119	CTAGGGATCACAATTATGGAAAG	Sequencing P_{BAD}*dcas9*
SJ_XT121	AAAGCGGGACCAAAGCCATGA	PCR check P_{BAD}*dcas9* forward
SJ_XT122	GGTTAAGATAAAACCTGCCTGC	PCR check P_{BAD}*dcas9* reverse
SJ_XT123	GCGATTGGCAAAACCATCTGA	Sequencing P_{BAD}*dcas9*
SJ_XT130	ACCAGGCCAGCCACGTTTCTGCGAAAACGCGGGAAAAAGTGGAAGCGGCGTCCTAATTTTTGTTGACACTCTATC	make *tet-sacB<>lacI* forward
SJ_XT131	AAGCCTGGGGTGCCTAATGAGTGAGCTAACTCACATTAATTGCGTTGCGCATCAAAGGGAAAACTGTCCATATGC	make *tet-sacB<>lacI* reverse

SJ_XT132	TTTCTGCGAAAACGCGGGAAAAGTGGAAGCGGCGGCGCAACGCAATTAATGTGAGTTAGCTCACTCATT	make *tet-sacB<>lacI* deletion
SJ_XT133	CTATTCTGGTGGCCGGAAGG	check *tet-sacB<>lacI* deletion forward
SJ_XT134	TCTTCGCTATTACGCCAGCT	check *tet-sacB<>lacI* deletion reverse
SJ_XT136	ACCGACATATTTGCAACTCAATATTCACAACAACCAATTCTAAAGATCTTTGACAGCTAGCTCAGTCCTAGGTATAATGCTAGCAACTTT	make *GalM_tet-sacB*<>pgRNA*_gpmA* forward
SJ_XT137	CAACAGCAATGCTTACGCATAACCATAGCGAAAATAGTGGCGCAGTGTAACTGCAGTCTAGACTCGAGTAAG	make *GalM_tet-sacB*<>pgRNA*_gpmA* reverse
SJ_XT138	GGCAAGAAAGTGGCGGCGCATGTC	check *GalM_tet-sacB*<>pgRNA*_gpmA* forward
SJ_XT139	CCATGACGAACCAGAACCAG	check *GalM_tet-sacB*<>pgRNA*_gpmA* reverse
SJ_XT140	CAATTCTAAAGATCTTTGACAGCTAGCTCAGTCCTAGGTATAATGCTAGCTCCTAATTTTTGTTGACACTCTATC	Make *GalM*_promoter-*tet-sacB*-handle-terminater_*gpmA* forward
SJ_XT141	GTTGATAACGGACTAGCCTTATTTTAACTTGCTATTTCTAGCTCTAAAACATCAAAGGGAAAACTGTCCATATGC	Make *GalM*_promoter-*tet-sacB*-handle-terminater_*gpmA* reverse
SJ_XT142	TTGACAGCTAGCTCAGTCCTAGGTATAATGCTAGCgtagtgacaagtgttggccaGTTTTAGAGCTAGAAATAGCAAGTTAAAATAAGGC	make *yfp* sgRNA
SJ_XT156	CGCTTTTTATCGCAACTCTCTACTGTTTCTCCATACCCGTTTTTTTGGATAGGAGGATGAAACGATGAGTAAAGGTGAAGAACTGTTCAC	make *msfGFP<>araBAD* forward
SJ_XT157	TGAgTATAGCCTGGTTTCGTTTGATTGGCTGTGGTTTTATACAGTCATTATTTGTAGAGTTCATCCATGCC	make *msfGFP<>araBAD* reverse
SJ_XT158	AATCAGTTTGTTTTCTGGCTGGGCTCTGGCTGTTGTCTCATCCTCGCCGTTTTACTCTTTTTCGCCAAAA	make *lacY* A177C
SJ_XT185	TTGACAGCTAGCTCAGTCCTAGGTATAATGCTAGCAGATGTTCTTCAGCCGCGTTGTTTTAGAGCTAGAAATAGCAAGTTAAAATAAGGC	*lexA* sgRNA
SJ_XT186	TTGACAGCTAGCTCAGTCCTAGGTATAATGCTAGCTTTACGCAGCGCTTGTGCATGTTTTAGAGCTAGAAATAGCAAGTTAAAATAAGGC	Make *ftsZ* sgRNA
SJ_XT245	TTGACAGCTAGCTCAGTCCTAGGTATAATGCTAGCCGCCACGGATTTGACATTCCGTTTTAGAGCTAGAAATAGCAAGTTAAAATAAGGC	Make *rpoB* sgRNA
SJ_XT246	TTGACAGCTAGCTCAGTCCTAGGTATAATGCTAGCAGGACGGGGTTTTCTCGTTGGTTTTAGAGCTAGAAATAGCAAGTTAAAATAAGGC	Make *dnaG* sgRNA
SJ_XT248	TTGACAGCTAGCTCAGTCCTAGGTATAATGCTAGCGTTTTAGAGCTAGAAATAGCAAGTTAAAATAAGGC	make no sgRNA control
SJ_XT293	TTGACAGCTAGCTCAGTCCTAGGTATAATGCT	make *lacZ* sgRNA.

	AGCACGACGACAGTATCGGCCTCGTTTTAGAG CTAGAAATAGCAAGTTAAAATAAGGC	
SJ_XT311	AGCCGGATTATCTGGATATCCCAGCATTCCTG CGTAAGCAAGCTGATtaaTTTTGGATAGGAGGA TGAAACG	make *ftsZ*-SD-*msfGFP-cm* forward
SJ_XT312	GCGGGCCAGTTTAGCACAAAGAGCCTCGAAA CCCAAATTCCAGTCAATTCACCAGCAATAGAC ATAAGCGGC	make *ftsZ*-SD-*msfGFP-cm* reverse
SJ_XT317	TGAAAGAGATTCGTTCGCTGGGTATCAACATC GAACTGGAAGACGAGtaaTTTTGGATAGGAGGA TGAAACGATG	make *rpoB*-SD-*msfGFP-cm* forward
SJ_XT318	AATCCGCTGCCGGGTTTTAACCCGACAGCAGT GACCTGTTTGAGCGAGAAACCAGCAATAGAC ATAAGCGGC	make *rpoB*-SD-*msfGFP-cm* reverse
SJ_XT320	AAGAACGCCTGGAGCTCTGGACATTAAACCA GGAGCTGGCGAAAAAGtgaTTTTGGATAGGAG GATGAAACGATG	make *dnaG*-SD-*msfGFP-cm* forward
SJ_XT321	TGCGGCTGTCGGGGGCTTCCCGATCGCTCTTC GGCACTTAAGCCGTTAAAACCAGCAATAGAC ATAAGCGGC	make *dnaG*-SD-*msfGFP-cm* reverse
SJ_XT323	CCATTGAAGGGCTGGCGGTTGGGGTTATTCGC AACGGCGACTGGCTGtaaTTTTGGATAGGAGGA TGAAACGATG	make *lexA*-SD-*msfGFP-cm* forward
SJ_XT324	GATGAAAAACAAACCGCGACGCCAGGCGGCA TCGCGGTCTCAGAGATATGACCAGCAATAGAC ATAAGCGGC	make *lexA*-SD-*msfGFP-cm* reverse
SJ_XT326	AGCTGAGCGCCGGTCGCTACCATTACCAGTTG GTCTGGTGTCAAAAAtaaTTTTGGATAGGAGGA TGAAACGATG	make *lacZ*-SD-*msfGFP-cm* forward
SJ_XT327	ATGGATTTCCTTACGCGAAATACGGGCAGACA TGGCCTGCCCGGTTATTAACCAGCAATAGACA TAAGCGGC	make *lacZ*-SD-*msfGFP-cm* reverse
SJ_XT339	aatttatttgcactactggaaaactacctgttccaTGGCCTACACTT GTCACTACtttcggttatggtctaaaatgctttgctagatacc	make *yfp* mutation_1
SJ_XT340	aatttatttgcactactggaaaactacctgttccaTGGCCTACTCTT GTCACTACtttcggttatggtctaaaatgctttgctagatacc	make *yfp* mutation_2
SJ_XT341	aatttatttgcactactggaaaactacctgttccaTGGCCTACTCTA GTCACTACtttcggttatggtctaaaatgctttgctagatacc	make *yfp* mutation_3
SJ_XT342	aatttatttgcactactggaaaactacctgttccaTGGCCTACTCTA GTGACTACtttcggttatggtctaaaatgctttgctagataccc	make *yfp* mutation_4
SJ_XT343	aatttatttgcactactggaaaactacctgttccaTGGCCTACTCTA GTGACAACtttcggttatggtctaaaatgctttgctagatacc	make *yfp* mutation_5
SJ_XT347	gattctattaacaagggtatcacc	check *yfp*
SJ_XT348	atgcgtaaaggagaagaacttttcac	check *yfp*

Table S3. Genes not covered by the whole-genome sgRNA design screen.

**gene**	**aa encoded**	**function/family annotation**
ykgP	29 aa	putative oxidoreductase pseudogene
xisD	74 aa	DLP12 prophage pseudogene
exoD	92 aa	DLP12 prophage pseudogene
peaD	103 aa	DLP12 prophage pseudogene
ybfI	49 aa	putative transcriptional regulator pseudogene
jayE	259 aa	e14 prophage pseudogene
rhsO	506 aa	rhsC-linked pseudogene
ykgS	68 aa	CP4-6 prophage pseudogene
lomR	131 aa	Rac prophage pseudogene
aaaE	377 aa	e14 prophage pseudogene
intK	60 aa	Qin prophage pseudogene
yfjV	388 aa	CP4-57 prophage pseudogene
yoeD	42 aa	CP4-44 prophage pseudogene
ylbI	31 aa	Rhs family pseudogene
oweS	235 aa	CPS-53/KpLE1 prophage pseudogene
yoeH	53 aa	CP4-44 prophage pseudogene
yibU	66 aa	rhsA-linked pseudogene
mokA	60 aa	hokB TA system pseudogene
ysaC	33 aa	unknown pseudogene
yrdE	131 aa	unknown pseudogene
yjdQ	53 aa	unknown pseudogene
ysaD	64 aa	putative glycoside catabolism system pseudogene

yibS	83 aa	rhsA-linked pseudogene
yibW	121 aa	rhsA-linked pseudogene

Table S4. Changes in gene expression for the subsets of off-target matches discussed in the text.

The target genes are highlighted in green.

Strain	MG1655 tCRISPRi ΔintS[yfp cat]								MG1655 tCRISPRi							
sgRNA	yfp				sacB				lexA				rpoB			
Gene Set	7nt no mis.		12nt ≤ 2 mis.		7nt no mis.		12nt ≤ 2 mis.		7nt no mis.		12nt ≤ 2 mis.		7nt no mis.		12nt ≤ 2 mis.	
	yfp	-0.863	yfp	-0.863	allB	-1.067	yhbE	-0.283	lexA	-0.443	allS	-0.568	rpoB	-0.513	rpoB	-0.513
	setA	-0.385	dmsD	-0.406	ldtE	-0.998	caiA	-0.186	eutB	-0.413	lexA	-0.443	ypeA	-0.269	yibB	-0.445
	ilvG	-0.173	uspE	-0.291	tsaD	-0.59	dppB	-0.126	yceH	-0.308	hypB	-0.364	ydjO	-0.144	yrdD	-0.29
	ycaI	-0.161	ilvG	-0.173	nirB	-0.493	bcsB	-0.094	ybfE	-0.251	yceH	-0.308	pepD	-0.117	ndh	-0.25
	msbA	-0.052	msbA	-0.052	bcsF	-0.407	patA	-0.055	rbsR	-0.221	ccmA	-0.267	menD	-0.094	amyA	-0.182
	ydjE	-0.04	ydjE	-0.04	ydcU	-0.386	cyaY	-0.041	ybcY	-0.201	yqhD	-0.188	fumA	-0.091	sdhA	-0.072
	yqjA	-0.017	yebZ	0.036	lpxM	-0.35	serS	0.153	arsB	-0.17	etk	-0.175	rlmI	-0.082	yraQ	0.032
	nfrB	0.063	rstA	0.039	dnaN	-0.32	sodA	0.173	nagE	-0.163	obgE	-0.144	panD	-0.022	mepH	0.048
	fmt	0.074	yghD	0.085	yagF	-0.289	modF	0.243	obgE	-0.144	cydA	-0.121	amiA	0.021	nepI	0.076
	ubiA	0.092	ubiA	0.092	csgE	-0.289	ygcP	0.313	osmC	-0.137	ycbZ	-0.119	sbcB	0.043	rssB	0.13
	ecpC	0.149	renD	0.097	glsB	-0.285	yaaJ	0.422	xseB	-0.119	rpoB	-0.106	ruvA	0.052	yjbI	0.16
	dfp	0.195	aegA	0.137	katG	-0.15	yddH	0.79	emtA	-0.102	fruA	-0.103	pepP	0.058	yggN	0.187
	yiiR	0.229	nadA	0.161	dsbD	-0.143			pcnB	-0.101	murD	-0.1	ybaL	0.06	opgH	0.207
			fes	0.229	folC	-0.133			rapA	-0.098	frlB	-0.091	serA	0.072	yidE	0.293
			intE	0.229	rpoC	-0.111			frlB	-0.091	fdoG	-0.089	ydbA	0.081	flgA	0.368
			insF1	0.257	ypjA	-0.104			lsrR	-0.091	mfd	-0.062	mscM	0.088	insG	0.44
			ygaQ	0.308	mlaB	-0.104			mpaA	-0.089	rplA	-0.049	yggS	0.141	rclB	0.509
					uvrA	-0.087			gpmM	-0.088	metC	-0.048	puuA	0.141		
					ftsH	-0.078			lldD	-0.047	yfeR	-0.042	xylE	0.185		
					prfC	-0.074			mutS	-0.045	flk	-0.039	ycaM	0.222		
					rlmI	-0.069			trmA	-0.037	dsbD	-0.036	yneO	0.236		
					deaD	-0.067			dsbD	-0.036	pfo	-0.032	nanC	0.333		
					metB	-0.066			nagC	-0.033	garD	-0.015	leuO	0.514		
					pykA	-0.062			pfo	-0.032	srlD	-0.015	lsrA	0.634		
					rluB	-0.049			nagZ	-0.031	ybaL	-0.006	yhcH	0.731		
					rhlE	-0.044			fnr	-0.028	bglA	0				
					yegE	-0.004			xdhA	-0.021	thiI	0.004				
					tig	-0.001			purB	-0.02	efp	0.029				
					yejO	0.049			garD	-0.015	ykfC	0.034				
					gpmM	0.073			srlD	-0.015	yicR	0.034				
					yeeX	0.081			hflK	-0.01	ybhS	0.034				
					metQ	0.1			tqsA	-0.004	kdpE	0.034				
					yjiT	0.11			nuoN	-0.001	nanM	0.034				
					rbbA	0.113			degQ	0.008	pyrL	0.034				
					mngB	0.158			adhE	0.01	tpiA	0.035				
					gspK	0.167			mscS	0.012	ybeZ	0.047				
					typA	0.2			creC	0.013	yoaA	0.048				
					flgE	0.313			sslE	0.028	sucA	0.071				
					ygcP	0.313			oppD	0.029	pbpC	0.08				
					yddH	0.79			insO	0.034	yegH	0.101				
					araJ	0.942			kdpE	0.034	dhaR	0.101				
									ybiX	0.034	frdA	0.127				
									yicR	0.034	katE	0.21				
									uhpA	0.034	dinB	0.259				
									mutL	0.039	eutL	0.386				
									livK	0.04	rpmB	0.511				
									gcvP	0.04						
									ybeZ	0.047						
									sodB	0.055						
									proV	0.064						
									yedE	0.079						
									pbpC	0.08						
									arnD	0.085						
									ypfJ	0.085						
									glcB	0.113						
									rsmJ	0.115						
									citB	0.288						
									yaaJ	0.292						
									thiQ	0.335						
									nirB	0.335						
									yqeB	0.335						
									mdtP	0.636						
									flgK	0.937						

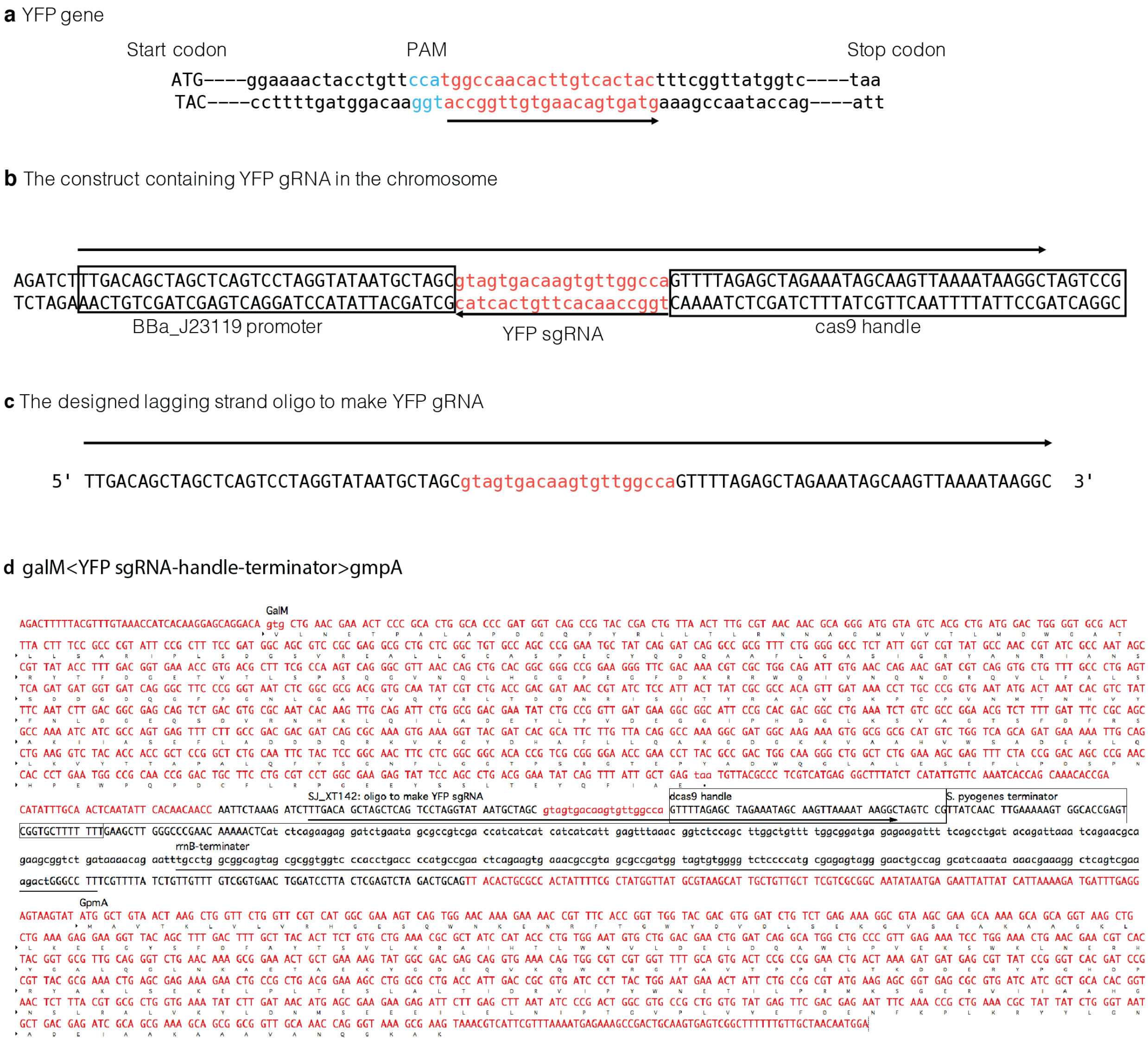

Figure S1

The 90nt lagging strand Oligo design for making YFP sgRNA

(a) The target region in the *yfp* gene. The Cyan letters indicate the PAM site the red letters are the *yfp* sgRNA target region. (b) The construct containing YFP sgRNA in the chromosome, BBa_J23119 promoter, YFP sgRNA and *cas9* handle are labeled. (c) The designed lagging strand oligo to make *yfp* sgRNA for the Non-template strand. d) The whole sequence between *galM* and *gpmA* in the chromosome.

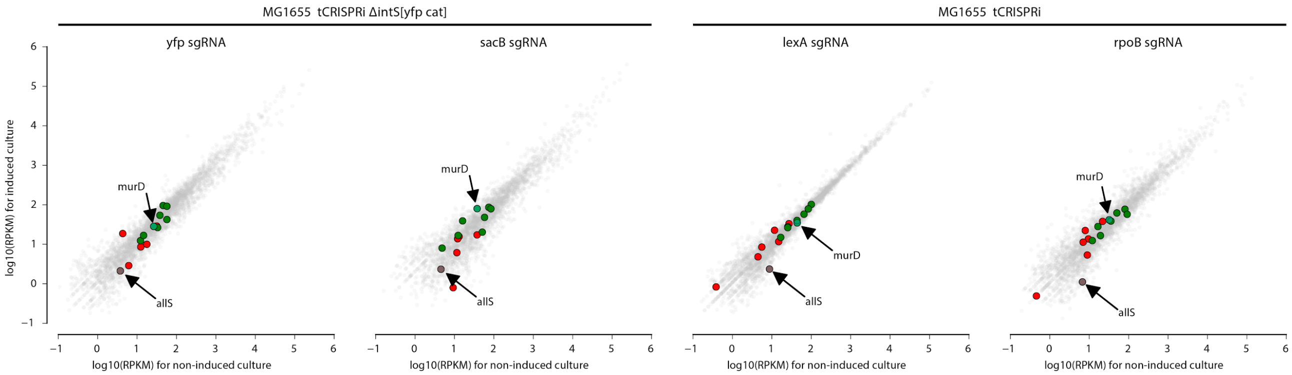

Figure S2.

Distribution of *all* (red circles) and *mur* genes (green circles) in log-log coplots of individual strains.

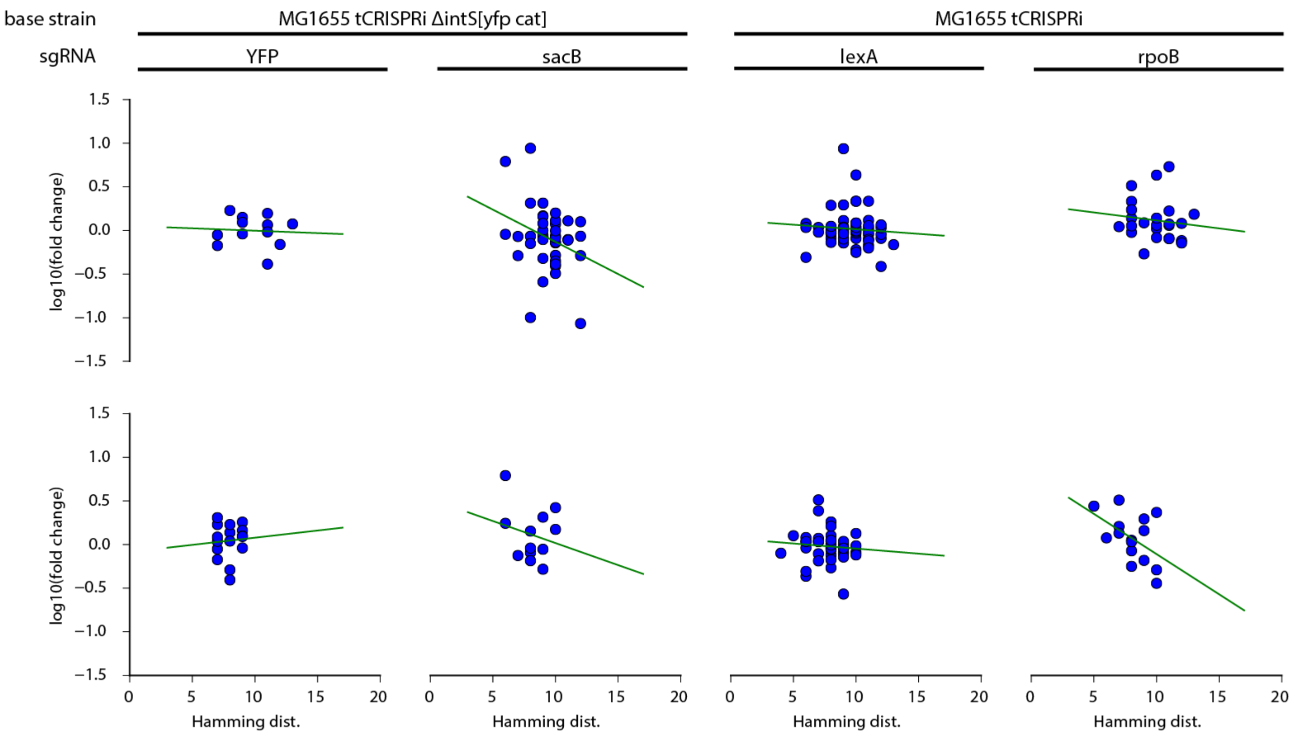

Figure S3.

Individual $\log_{10}$(fold change) and Hamming distance for genes identified as potential off-target matches to the sgRNA sequence. Top row, off-target perfect matches to the region I sgRNA sequence. Bottom row, off-target matches to the sgRNA sequence with a maximum Hamming distance of 10 regardless of mismatch position in sgRNA.

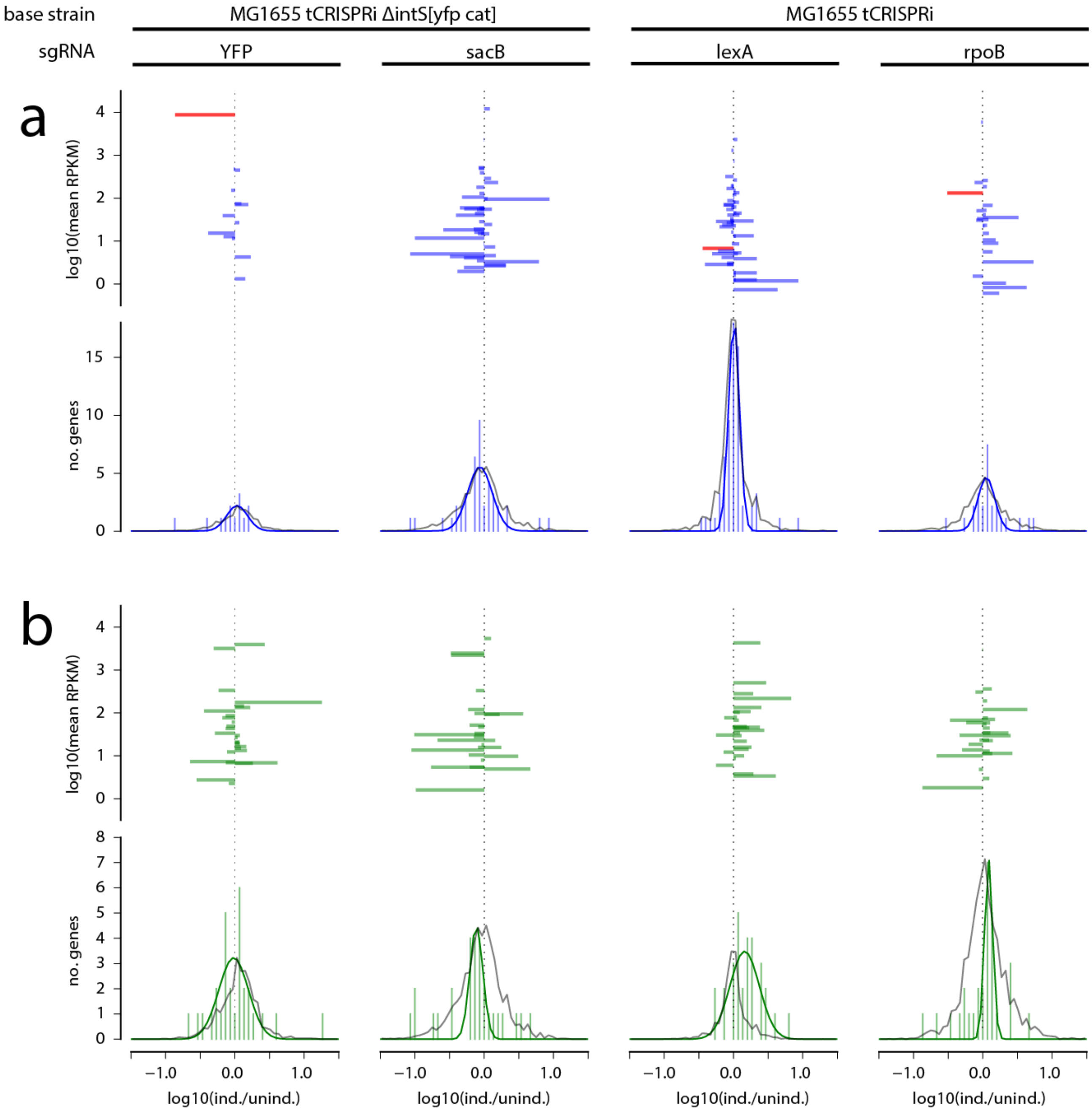

Figure S4.

Distributions of transcript abundance level changes with dCas9 induction for (a) potential off-target matches and (b) the *lexA* regulon for (from left to right) *yfp*, *sacB*, *lexA*, and *rpoB*. The skyline plots (top rows) show the individual genes (solid horizontal lines; target gene in red) and their changes versus no-change (dotted line). The histograms (bottom rows) show the binned data from the skyline plots (solid vertical lines), the fitted normal distributions of those bins (solid colored curved), and the global distributions of abundance level changes (grey curves, rescaled for proportion).

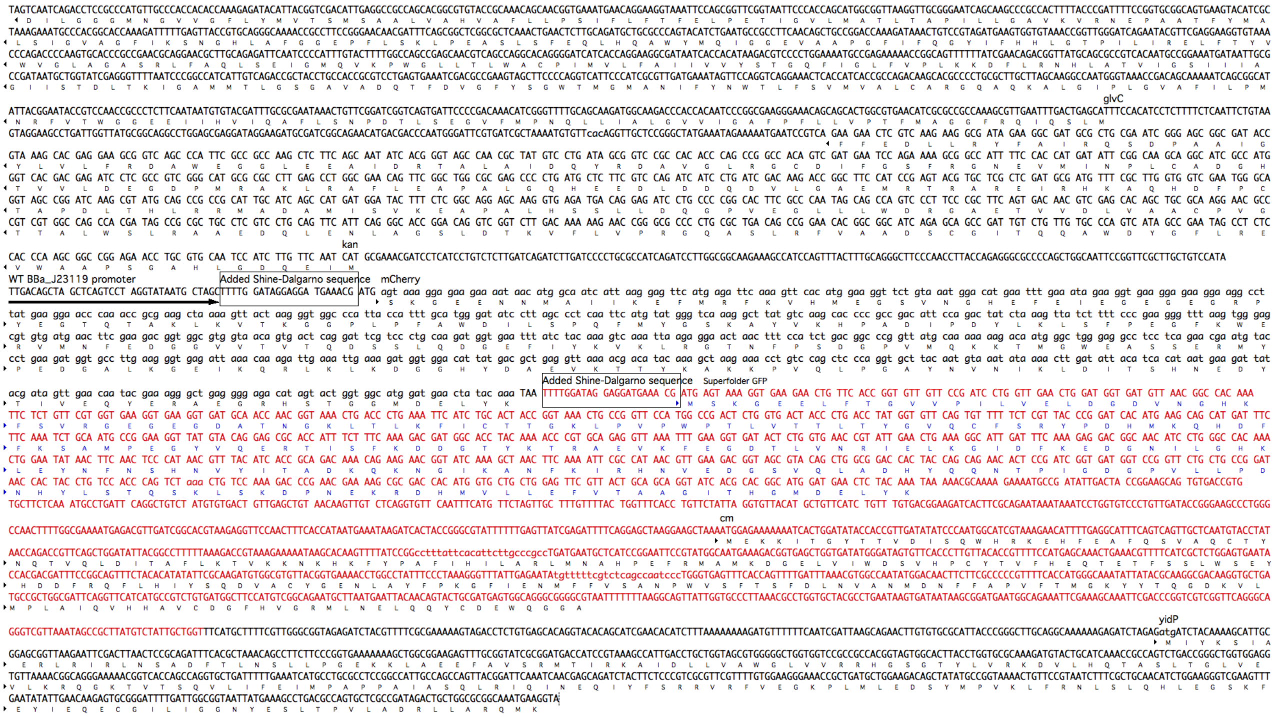

Figure S5

The construction of SJ_XTL410 (*mcherry* reporter strain). The *E.coli.* chromosomal gene *glvC* and *yidP* were indicated.The kanamycin resistant gene, pBBa_J23119 promoter, SD sequence, *mcherry* gene, *msfGFP* gene and chloramphenicol resistant gene were labeled. The red letters are the sequences amplified by PCR to insert into the downstream of the target gene stop codon.

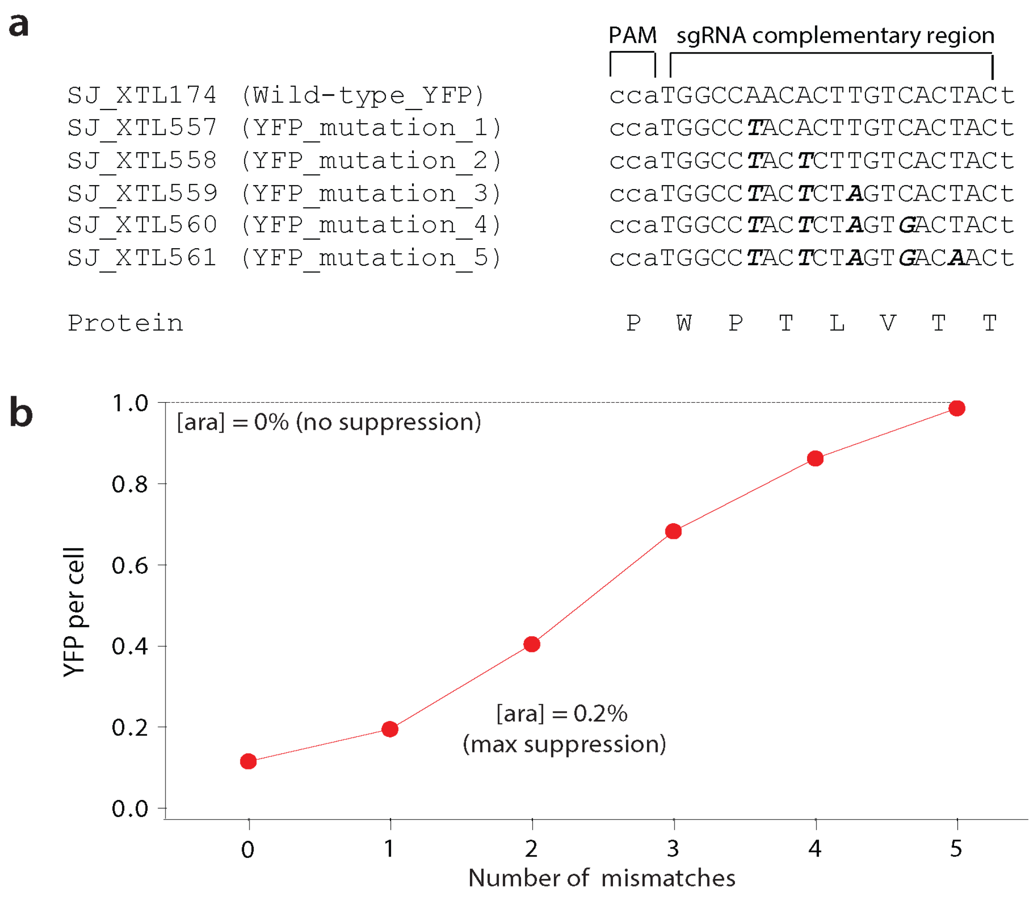

Figure S6.

Knockdown by tCRISPRi in the presence of sequence mismatches. (a) We generated wobble mutations in the coding sequence of the *yfp* gene. The bold Italic bases are the wobble mutations in YFP gene. (b) Knockdown of *yfp* by maximum suppression by tCRISPRi in the presence of sequence mismatches.

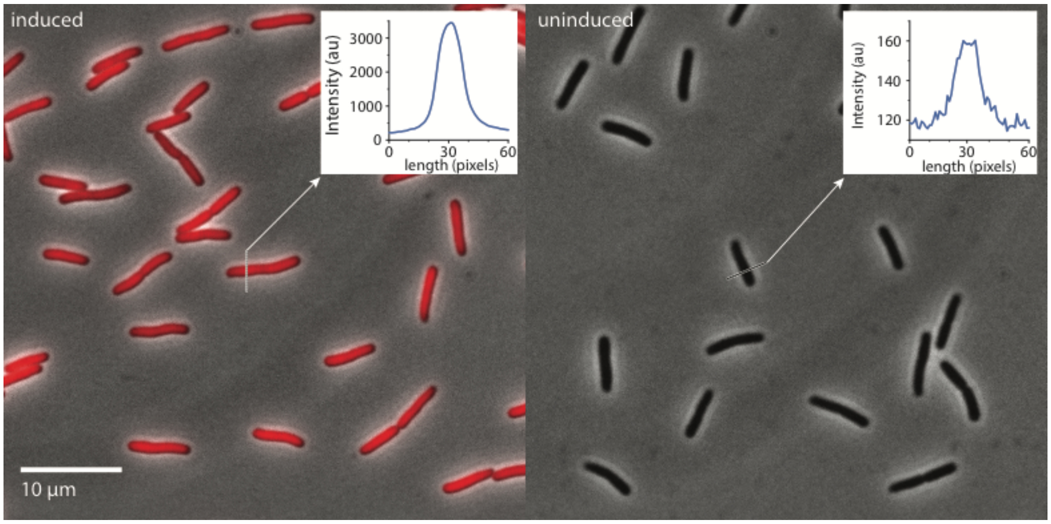

Figure S7.

Testing the Ptet promoter in a tCRISPRi strain SJ_XTL174 in MOPS rich glycerol medium. Plasmid encoding red fluorescent protein (RFP) under the Ptet promoter[31] was transformed to SJ_XTL174. During exponential growth, cell culture was transferred to two tubes. One of the tubes contained tetracycline (final concentration 0.1%) as a positive control and the other tube did not contain the inducer as a negative control. Cells were dilute 1000-fold and were grown for additional 2.5 hours, fixed, and imaged. The fluorescence level of the positive control was more than 20-fold higher than the negative control, confirming the expected behavior of the pTtet promoter in our tCRISPRi strain.

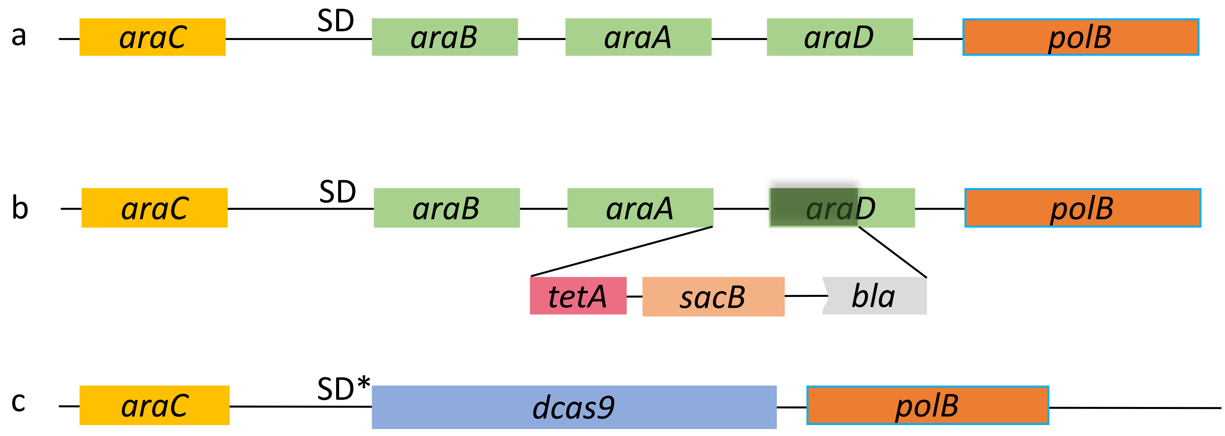

Figure S8

Construction of dCas9 controlled by the arabinose promoter P_{BAD}. (a) Wildtype *araBAD* operon with the *polB* gene of DNA Pol II downstream. SD indicates Shine-Dalgarno ribosome binding site. **(b)** Insertion of *tet-sacB bla* cassette downstream of *araA* and removing part of *araD*. This is strain XTL298 was described[28]. **(c)** Replacement of SD *araB, araA, cat, tetA, bla* and remainder of *araD* with the *dcas9* gene and an optimized SD* ribosome binding site

AAGTGTCTATAATCACGGCAGAAAAGTCCACATTGATTATTTGCACGGCGTCACACTTTGCTATGCCATAGCATTTTTATCCATAAGATTAGCGGATCCTACCTG
TTCACAGATATTAGTGCCGTCTTTTCAGGTGTAACTAATAAACGTGCCGCAGTGTGAAACGATACGGTATCGTAAAAATAGGTATTCTAATCGCCTAGGATGGAC

SJ_XT110 pBAD start **SD*** dCas9 D10

ACGCTTTTTATCGCAACTCTCTACTGTTTCTCCATACCCGTTTTTTTGGATAGGAGGATGAAACG ATG GAT AAG AAA TAC TCA ATA GGC TTA GCT
TGCGAAAAATAGCGTTGAGAGATGACAAAGAGGTATGGGCAAAAAAACCTATCCTCCTACTTTGC TAC CTA TTC TTT ATG AGT TAT CCG AAT CGA
M D K K Y S I G L A

ATC GGC ACA AAT AGC GTC GGA TGG GCG GTG ATC ACT GAT GAA TAT AAG GTT CCG TCT AAA AAG TTC AAG GTT CTG GGA
TAG CCG TGT TTA TCG CAG CCT ACC CGC CAC TAG TGA CTA CTT ATA TTC CAA GGC AGA TTT TTC AAG TTC CAA GAC CCT
I G T N S V G W A V I T D E Y K V P S K K F K V L G

AAT ACA GAC CGC CAC AGT ATC AAA AAA AAT CTT ATA GGG GCT CTT TTA TTT GAC AGT GGA GAG ACA GCG GAA GCG ACT
TTA TGT CTG GCG GTG TCA TAG TTT TTT TTA GAA TAT CCC CGA GAA AAT AAA CTG TCA CCT CTC TGT CGC CTT CGC TGA
N T D R H S I K K N L I G A L L F D S G E T A E A T

CGT CTC AAA CGG ACA GCT CGT AGA AGG TAT ACA CGT CGG AAG AAT CGT ATT TGT TAT CTA CAG GAG ATT TTT TCA AAT
GCA GAG TTT GCC TGT CGA GCA TCT TCC ATA TGT GCA GCC TTC TTA GCA TAA ACA ATA GAT GTC CTC TAA AAA AGT TTA
R L K R T A R R R Y T R R K N R I C Y L Q E I F S N

GAG ATG GCG AAA GTA GAT GAT AGT TTC TTT CAT CGA CTT GAA GAG TCT TTT TTG GTG GAA GAA GAC AAG AAG CAT GAA
CTC TAC CGC TTT CAT CTA CTA TCA AAG AAA GTA GCT GAA CTT CTC AGA AAA AAC CAC CTT CTT CTG TTC TTC GTA CTT
E M A K V D D S F F H R L E E S F L V E E D K K H E

CGT CAT CCT ATT TTT GGA AAT ATA GTA GAT GAA GTT GCT TAT CAT GAG AAA TAT CCA ACT ATC TAT CAT CTG CGA AAA
GCA GTA GGA TAA AAA CCT TTA TAT CAT CTA CTT CAA CGA ATA GTA CTC TTT ATA GGT TGA TAG ATA GTA GAC GCT TTT
R H P I F G N I V D E V A Y H E K Y P T I Y H L R K

AAA TTG GTA GAT TCT ACT GAT AAA GCG GAT TTG CGC TTA ATC TAT TTG GCC TTA GCG CAT ATG ATT AAG TTT CGT GGT
TTT AAC CAT CTA AGA TGA CTA TTT CGC CTA AAC GCG AAT TAG ATA AAC CGG AAT CGC GTA TAC TAA TTC AAA GCA CCA
K L V D S T D K A D L R L I Y L A L A H M I K F R G

CAT TTT TTG ATT GAG GGA GAT TTA AAT CCT GAT AAT AGT GAT GTG GAC AAA CTA TTT ATC CAG TTG GTA CAA ACC TAC
GTA AAA AAC TAA CTC CCT CTA AAT TTA GGA CTA TTA TCA CTA CAC CTG TTT GAT AAA TAG GTC AAC CAT GTT TGG ATG
H F L I E G D L N P D N S D V D K L F I Q L V Q T Y

AAT CAA TTA TTT GAA GAA AAC CCT ATT AAC GCA AGT GGA GTA GAT GCT AAA GCG ATT CTT TCT GCA CGA TTG AGT AAA
TTA GTT AAT AAA CTT CTT TTG GGA TAA TTG CGT TCA CCT CAT CTA CGA TTT CGC TAA GAA AGA CGT GCT AAC TCA TTT
N Q L F E E N P I N A S G V D A K A I L S A R L S K

TCA AGA CGA TTA GAA AAT CTC ATT GCT CAG CTC CCC GGT GAG AAG AAA AAT GGC TTA TTT GGG AAT CTC ATT GCT TTG
AGT TCT GCT AAT CTT TTA GAG TAA CGA GTC GAG GGG CCA CTC TTC TTT TTA CCG AAT AAA CCC TTA GAG TAA CGA AAC
S R R L E N L I A Q L P G E K K N G L F G N L I A L

TCA TTG GGT TTG ACC CCT AAT TTT AAA TCA AAT TTT GAT TTG GCA GAA GAT GCT AAA TTA CAG CTT TCA AAA GAT ACT
AGT AAC CCA AAC TGG GGA TTA AAA TTT AGT TTA AAA CTA AAC CGT CTT CTA CGA TTT AAT GTC GAA AGT TTT CTA TGA
S L G L T P N F K S N F D L A E D A K L Q L S K D T

TAC GAT GAT GAT TTA GAT AAT TTA TTG GCG CAA ATT GGA GAT CAA TAT GCT GAT TTG TTT TTG GCA GCT AAG AAT TTA
ATG CTA CTA CTA AAT CTA TTA AAT AAC CGC GTT TAA CCT CTA GTT ATA CGA CTA AAC AAA AAC CGT CGA TTC TTA AAT
Y D D D L D N L L A Q I G D Q Y A D L F L A A K N L

TCA GAT GCT ATT TTA CTT TCA GAT ATC CTA AGA GTA AAT ACT GAA ATA ACT AAG GCT CCC CTA TCA GCT TCA ATG ATT
AGT CTA CGA TAA AAT GAA AGT CTA TAG GAT TCT CAT TTA TGA CTT TAT TGA TTC CGA GGG GAT AGT CGA AGT TAC TAA
S D A I L L S D I L R V N T E I T K A P L S A S M I

AAA CGC TAC GAT GAA CAT CAT CAA GAC TTG ACT CTT TTA AAA GCT TTA GTT CGA CAA CAA CTT CCA GAA AAG TAT AAA
TTT GCG ATG CTA CTT GTA GTA GTT CTG AAC TGA GAA AAT TTT CGA AAT CAA GCT GTT GTT GAA GGT CTT TTC ATA TTT
K R Y D E H H Q D L T L L K A L V R Q Q L P E K Y K

Figure S9

Sequence of dCas9 Expression Construct. The *dcas9* gene sequence is shown (blue) with the protein sequence below. DNA codon changes that create the defective cleavage mutant are labeled in red. The long blue arrows indicate the sequences of the single strand oligo primers used to amplify by PCR the *dcas9* gene from the plasmid DNA pdCas9[12].These primers had at their 5' end homology to the upstream and downstream regions of the *ara* operon generating an amplified *dcas9* gene flanked by these homologies. The upstream homology sequence (red) included the P_{BAD} promoter and the modified SD* to optimize translation initiation. The downstream homology sequence (black) included the *araD* stop codon (TAA) and region beyond the *ara* operon. This PCR product was recombined with strain SJ_XTL 175 containing the *tet-sacB-bla* (Table S1). The sucrose resistant (Cm^S and Ap^S) recombinants had inserted *dcas9* under P_{BAD} control. The black bar represents the optimized sequence of the Shine-Dalgarno (SD*) to enhance translation of dcas9. A partial sequence of the *polB* gene downstream of the *ara* operon is shown.
